# Recent Developments in Plastic Deformation Behavior of Titanium and Its Alloys During the Rolling Process: A Review

**DOI:** 10.3390/ma17246060

**Published:** 2024-12-11

**Authors:** Donghee Ryu, Yulhee Kim, Sahn Nahm, Leeseung Kang

**Affiliations:** 1 Korea National Institute of Rare Metals, Korea Institute of Industrial Technology, Incheon 21655, Republic of Korea; rdh1126@kitech.re.kr (D.R.); dbfgml1116@kitech.re.kr (Y.K.); 2 Department of Materials Science and Engineering, Korea University, Seoul 02841, Republic of Korea; snahm@korea.ac.kr

**Keywords:** titanium, plastic deformation, rolling process, microstructure analysis, mechanical properties

## Abstract

Titanium (Ti) and its alloys are used in various applications, including aircraft frames, ship parts, heat exchangers, and evaporator tubes, because of their extraordinary properties, such as high specific strength, excellent corrosion resistance at high temperatures, good castability, and weldability. Plastic deformation plays a crucial role in securing the appropriate microstructure and strength of Ti and alloys in these applications. The rolling process, one of the most useful methods for plastic deformation, causes efficient deformation inside the materials, resulting in grain refinement, dislocation slip, and twinning. Recent studies on the rolling behaviors of Ti and its alloys have explored their crystallographic and mechanical properties. These investigations primarily analyzed the microstructural changes and their influence on the mechanical properties under different temperatures and rolling methods. This study elucidates a complex relationship between the processing conditions and the resulting properties. Therefore, this paper presents a comprehensive review of the state-of-the-art Ti rolling. Various key aspects for verifying the microstructure of Ti and its alloys are discussed, including electron backscatter diffraction analysis, Schmidt factor, and misorientation distribution.

## 1. Introduction

Rapid advancements in the aerospace, automotive, marine, and medical industries have increasingly emphasized the importance of developing new materials to meet diverse demands. New materials are employed in the aerospace sector for aircraft fuselage components, engine parts, and fuel systems for rockets and satellites, where their mechanical properties are required to be maintained at extreme temperatures while being lightweight and highly corrosion resistant [1,2,3,4,5,6,7,8,9,10]. In the automotive industry, reducing the inertial mass, friction, and noise in engine parts, connecting rods, valves, and springs is critical for improving fuel efficiency, thus making lightweight and corrosion-resistant materials essential [11,12,13,14,15,16,17,18,19,20,21]. In the marine industry, offshore equipment and ship manufacturing require materials with high tensile and compressive strength, corrosion resistance, and fatigue strength to perform well in deep-sea environments [22,23,24,25,26,27,28,29]. In the biomedical sector, implants, artificial bones, artificial joints, and surgical devices necessitate nontoxicity and corrosion resistance to prevent degradation or leaching of components into the body during prolonged internal use while effectively replacing biological tissues [30,31,32,33,34,35,36,37,38,39,40,41]. Titanium (Ti) is a material that meets the diverse demands of various industries owing to its high density, strength, and corrosion resistance. Consequently, research efforts have intensified to develop Ti compositions and improve their properties tailored to the specific requirements of each application.

To understand why Ti exhibits such beneficial properties, examining its characteristics from a material engineering perspective is essential. Ti typically has two crystalline structures: body-centered cubic (BCC) and hexagonal close-packed (HCP). In the BCC structure, eight Ti atoms form a single lattice, whereas in the HCP structure, six atoms form a hexagonal lattice surrounded by 12 neighboring atoms. Ti usually stabilizes in the HCP structure (α-Ti) at room temperature [42,43,44,45]. However, the addition of stabilizing elements for the beta phase, such as vanadium (V), niobium (Nb), tantalum (Ta), and molybdenum (Mo), along with heat treatment above 882 °C, leads to the formation of the BCC phase (β-Ti) [46,47,48]. Thus, Ti generally exhibits an HCP structure below 882 °C. Compared to the BCC structure, the HCP structure provides slightly lower strength but features lower density and enhanced ductility. The mechanical properties of HCP differ from those of BCC because of the c/a ratio, which represents the height of the cell along the c-axis relative to its width along the a-axis. Among the elements with HCP structures, zirconium, magnesium, lead, and Ti exhibit c/a ratios of 1.595 [49], 1.624 [49], 1.856 [50], and 1.587 [49], respectively. The relatively low c/a ratio of Ti contributes to its high thermal conductivity, low thermal expansion coefficient, and excellent strength and ductility.

**Figure 1:**
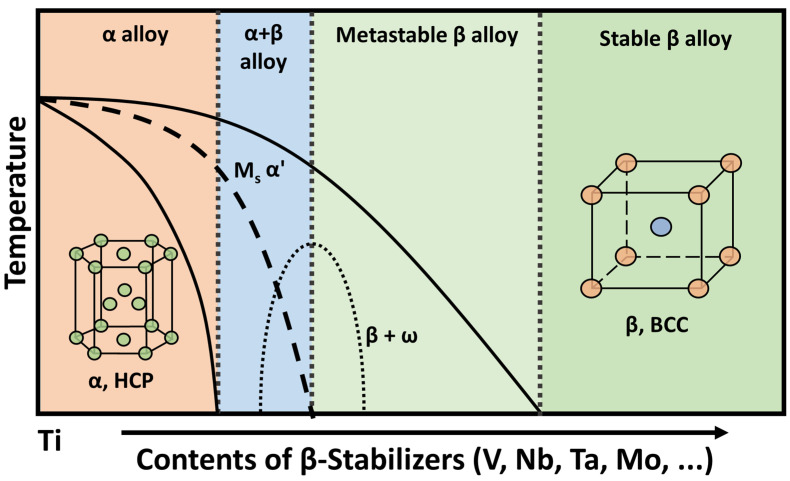
Ti alloy phase diagram depending on temperature and β-stabilizer content.

Based on their crystalline structures, Ti alloys are classified into five major groups: α, near-α, α + β, near-β, and β alloys (Figure 1), depending on their microstructures and stabilizing elements. Each of these groups has distinctive phase compositions and mechanical properties [51,52]. α alloys, primarily composed of commercially pure Ti (CP-Ti) or stabilized by Al (aluminum) and oxygen, exhibit a stable single-phase HCP structure at room temperature. These alloys are characterized by exceptional thermal stability, high creep resistance, and low ductility. Near-α alloys consist of two phases. One is an α-dominated microstructure, which enhances workability without compromising thermal performance. The other is a minor fraction of β-phase, stabilized by β-stabilizers such as molybdenum or zirconium. The presence of β-phase significantly improves both the toughness and strength of near-α alloys. α + β alloys, exemplified by Ti-6Al-4V, achieve a balance among ductility, corrosion resistance, and strength due to the coexistence of α and β phases. Their microstructure can be modified through heat treatment and thermo-mechanical processing to control the morphology and size of grains, and phase distribution. These modifications enable the enhancement of key properties such as strength, fatigue resistance, and toughness. Near-β alloys, which contain a higher proportion of β-phase due to the presence of strong β-stabilizers such as vanadium, niobium, or tantalum, retain a stable β-phase at room temperature. These alloys exhibit enhanced hardenability and strength through heat treatment; however, their BCC structure renders them more brittle at cryogenic temperatures compared to α-dominated alloys. β alloys, fully stabilized in the β-phase through significant additions of β-stabilizers, exhibit a highly process-dependent microstructure. Metastable phases, such as martensite (α’) or ω-phase, can form during rapid cooling. Martensite formation increases strength but may also induce brittleness, while the ω-phase typically reduces ductility, highlighting the importance of precise control during processing. The interplay between stable and metastable phases, influenced by factors such as cooling rates, alloying elements, and thermo-mechanical conditions, determines critical attributes like grain size, phase distribution, and texture. This dynamic behavior enables the design of Ti alloys optimized for specific mechanical and structural requirements.

In most cases, Ti and its alloys undergo plastic deformation to further enhance their properties. Plastic deformation is a critical manufacturing process for improving the mechanical properties of materials and is an effective method for significantly enhancing the strength and hardness of metallic materials. Plastic deformation enables materials to acquire new properties absent in their initial state, significantly enhancing their applicability. Specifically, plastic deformation can lead to substantial increases in the yield strength (YS) by promoting grain refinement and increasing the dislocation density within the material. As the grains become finer, the grain boundaries hinder dislocation movement, thus increasing the resistance to deformation and enhancing the strength of the material [53]. An increased dislocation density further improves strength by facilitating interactions among dislocations, which bolsters material durability and optimizes its ability to withstand high-temperature and high-pressure environments [54]. In addition, plastic deformation enhances workability, allowing for the formation of complex shapes. During this process, the material deforms into the desired shape under external forces, reorganizing its internal structure to exhibit the optimized properties. This is essential for producing high-value-added products and plays a crucial role in industries such as aerospace, automotive, and electronics, where lightweight and high-performance materials are in high demand. Plastic deformation is a manufacturing process that shapes metallic materials through deformation during processing, encompassing procedures such as forging, extrusion, drawing, and rolling. Rolling is a process in which metallic materials are passed between rotating rolls to reduce their thickness or change their shape.

Rolling is primarily used in the production of sheets and bars, categorized into hot rolling and cold rolling. Hot rolling is performed above the recrystallization temperature to improve the ductility and workability of the material, whereas cold rolling is conducted at room temperature to provide higher strength and dimensional accuracy. The plastic deformation during the rolling process alters the grain size and rearranges the microstructure, hence enhancing the mechanical properties of the final product. Therefore, the rolling process plays a crucial role in shaping and controlling the properties of metallic materials, particularly in the industrial applications of high-performance metals such as Ti and its alloys.

After the rolling process, appropriate heat treatments can be applied to enhance the mechanical properties and optimize the microstructure. Heat treatments, such as annealing and post-rolling recrystallization, are employed to relax residual stresses generated during rolling, refine the grain structure, and enhance the material’s ductility and toughness. The temperature and duration of heat treatment are determined by the initial microstructure and the targeted mechanical properties. Furthermore, the potential formation of adiabatic shear bands during the rolling process must be carefully considered [55]. Adiabatic shear bands are localized regions of intense plastic deformation, where adiabatic heating occurs, potentially resulting in the formation of ultrafine grains or even amorphous regions. The formation of adiabatic shear bands is influenced by factors such as deformation temperature, strain rate, and material properties. These shear bands are typically observed in high-strain regions where the strain rates surpass the material’s ability to dissipate heat efficiently, leading to localized softening and failure. In Ti, the presence of adiabatic shear bands can significantly alter the microstructure, texture, and mechanical properties, making it essential to control their formation during the rolling process.

The rolling process plays a crucial role in modifying the microstructure and mechanical properties of each Ti alloy group [56,57,58]. In α alloys, rolling induces significant changes in grain size and texture. This, in turn, enhances strength and ductility due to the recrystallization and/or strain hardening. However, due to their limited phase stability, the α phase requires careful management during rolling to optimize mechanical performance. In near-α alloys, rolling refines the β phase, improving both workability and mechanical strength while maintaining thermal stability. In α + β alloys, rolling enables precise control of the microstructure by refining both the α and β phases, thereby optimizing properties such as strength, fatigue resistance, and toughness. For near-β alloys, rolling significantly refines the β phase, resulting in enhanced strength and hardness. However, attentive control of the rolling process is necessary to prevent brittleness associated with the BCC structure at cryogenic temperatures. At last, β alloys undergo a martensitic transformation during rapid cooling after rolling, which increases strength but may reduce toughness. The rolling process for these alloys demands precise control over temperature and deformation to balance phase stability and mechanical performance. Across all Ti alloy groups, rolling is crucial for determining the grain structure, phase distribution, and texture, ultimately enhancing the alloys’ properties for various applications.

Therefore, this study aims to conduct an in-depth analysis of the physical and mechanical properties of Ti and Ti alloys that meet various requirements, including light weight, high strength, and high corrosion resistance, as well as the various rolling processes that can optimize these properties. First, an overview of plastic deformation and an introduction to the mechanisms of rolling are provided, followed by information on texture analysis methods that play a significant role in microstructural analysis. Different rolling processes are then categorized based on the rolling temperature and direction, and a multifaceted analysis is conducted on the microstructures and mechanical properties of Ti and its alloys, along with the effects of the plastic deformation techniques for each process. Finally, by introducing applications in which rolled Ti is primarily used, the properties of Ti required in these industries and potential directions for future improvements are discussed. By analyzing rolled Ti and Ti alloys from various perspectives, this study provides valuable information for the future development of Ti and its alloys to meet technical demands across diverse industrial fields.

## 2. Fundamentals of Ti Rolling

### 2.1. Plastic Deformation by Rolling

The rolling process is a crucial manufacturing operation in metal processing that involves passing the material between rotating rolls to reduce its thickness and achieve the desired shape. Rolling can be broadly classified into shape rolling and flat rolling, based on the final shape of the product. Shape rolling is performed to produce products with specific shapes, such as H-beams or I-beams. In contrast, flat rolling involves passing materials such as slabs, blooms, or billets produced by continuous casting through two rotating rolls to obtain thin, flat forms. During this process, the thickness decreases while the length increases, primarily resulting in flat products such as plates and coils.

**Figure 2:**
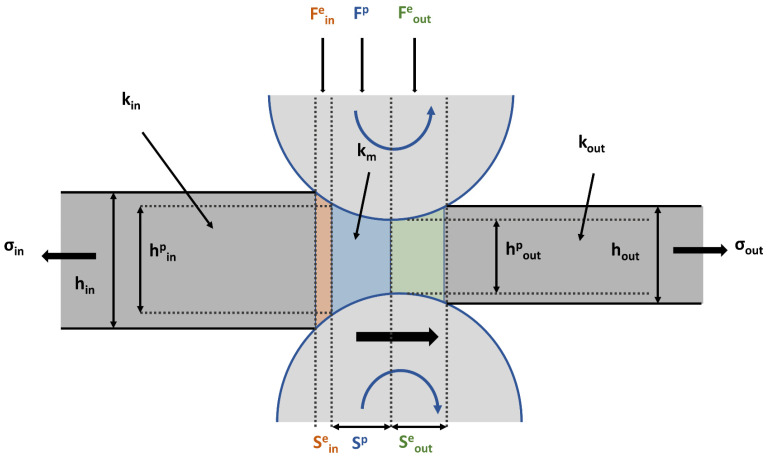
Schematic of force, thickness, deformation resistance, and stress associated with the deformation zones formed during the rolling process.

The rolling process involves the material entering between two independently driven rotating rolls, which exert a compressive force that reduces the thickness. Figure 2 illustrates the force, thickness, deformation resistance, and stress associated with the deformation zones formed during the rolling process. The compression zone regions of elastic and plastic deformation formed during the rolling process are as follows: $S_{in}^{e}$ is the entry elastic compression zone, $S^{p}$ is the plastic deformation zone, and $S_{out}^{e}$ is the exit elastic recovery zone. The forces acting in each zone are composed of the following deformation rolling forces: $F_{in}^{e}$ is the rolling force in the entry elastic deformation zone, $F^{p}$ is the rolling force in the plastic deformation zone, and $F_{out}^{e}$ is the rolling force in the exit elastic deformation zone. The thicknesses of the plates at the entry and exit sections are defined as follows: $h_{in}$ is the entry plate thickness, $h_{in}^{p}$ is the entry plate thickness in the plastic deformation zone, $h_{out}$ is the exit plate thickness, and $h_{out}^{p}$ is the exit plate thickness in the plastic deformation zone. The deformation resistance in each zone is represented as follows: $k_{in}$ is the entry plate resistance, $k_{m}$ is the average deformation resistance, and $k_{out}$ is the exit plate resistance. Additionally, $\sigma_{in}$ represents back tension, and $\sigma_{out}$ represents front tension.

(1) $$F=F^{e}+F^{p}$$

The rolling process must consider both elastic and plastic deformations of the material. During the rolling process, the material undergoes forces in the elastic compression zone ($S_{in}^{e}$), plastic deformation zone ($S^{p}$), and elastic recovery zone ($S_{out}^{e}$). The total load experienced by the material during this process is represented as the sum of the forces arising from elastic deformation ($F^{e}$) and plastic deformation ($F^{p}$), which can be expressed using the Bland–Ford and Hill equations (Equation (1)) [59,60].

(2) $$F^{e}=F_{in}^{e}+F_{out}^{e}=\frac{2}{3}\sqrt{\frac{1-v^{2}}{E}k_{m}\frac{h_{out}}{h_{in}-h_{out}}}(k_{m}-\xi )b\sqrt{R^{\prime }}(h_{in}-h_{out})$$

(3) $$k_{m}=B\;exp(\frac{A}{T+273})\varepsilon^{n}(\dot{\varepsilon }{)}^{m}$$

(4) $$\xi =\alpha \sigma^{in}+\beta \sigma^{out}$$

The force in the elastic deformation zone can be defined as the sum of the forces at the entry and exit, as expressed in Equation (2), where ν is the Poisson’s ratio, E is the Young’s modulus, ξ is the sum of the back tension and front tension, b is the plate width, and $R^{\prime }$ is the roll flattening radius. In Equation (3), which represents the average deformation degree ($k_{m}$), n, m, A, and B are tuning parameters for metal coil grades, $\dot{\varepsilon }$ is the strain rate that depends on time, r is the reduction, and μ is the friction coefficient. Equation (4) illustrates the sum of the back tension and front tension (ξ), where α is the influence coefficient at the back tension and β is the influence coefficient at the front tension. The term $\frac{1-v^{2}}{E}$ in Equation (2) represents the elastic deformation of the material, whereas $k_{m}\frac{h_{out}}{h_{in}-h_{out}}$ indicates how the deformation resistance during the rolling process is affected by the difference in the thickness of the material. As the thickness difference increases, the required deformation resistance also increases, highlighting the important role of the average deformation resistance $k_{m}$ in this process. The term $\left(k_{m}-\xi \right)$ signifies that the deformation resistance can be reduced under certain conditions, implying that the actual deformation resistance changes owing to the tension that naturally arises during the rolling process. Additionally, $b\sqrt{R^{\prime }}(h_{in}-h_{out})$ denotes the contact length between the material and roll during rolling. Therefore, the force in the elastic deformation zone is influenced by the inherent properties of the material, such as ν and E, as well as by the deformation resistance during the rolling process and the roll contact length.

(5) $$F^{p}=Q_{Fp}((k_{m}-\xi ))b\sqrt{R^{\prime }}(h_{in}-h_{out})$$

(6) $$Q_{Fp}=1.08-1.02r+1.79r\mu \sqrt{1-r}\sqrt{\frac{R^{\prime }}{h_{out}}}$$

The force in the plastic deformation zone is represented by Equation (5), where $Q_{Fp}$ denotes the influence coefficient of friction. In Equation (6), corresponding to $Q_{Fp}$, r represents the reduction, and μ indicates the friction coefficient. The deformation resistance term $\left((k_{m}-\xi ))b\sqrt{R^{\prime }}(h_{in}-h_{out}\right)$ associated with the contact length between the material and roll is similar to that in the elastic deformation zone. However, a key difference is that deformation in the elastic region occurs minimally, resulting in negligible relative sliding between the material and roll. Thus, the elastic restoring or compressive forces play a more significant role than friction. In contrast, in the plastic deformation zone, a considerable amount of sliding occurs, and the frictional force between the roll and material surface directly affects the internal deformation distribution and rolling force. Therefore, in the plastic deformation zone, the force is influenced by the reduction and friction coefficients, as well as by the contact length at which the material interacts with the roll.

(7) $$H_{delivery}=\frac{F}{M}+S$$

Equation (7) represents the relationship between the delivery thickness after the rolling process, considering the rolling load, mill modulus, and gap between the rollers. Here, $H_{delivery}$ stands for the delivery thickness, F for the rolling load, M for the mill modulus, and S for the gap between the rollers. Because M is an intrinsic value that reflects the mechanical properties of the rolling mill, it can be utilized in conjunction with the total force equations from the elastic deformation zone (Equation (2)) and plastic deformation zone (Equation (5), as shown in Equation (1). By adjusting the gap (S) during the rolling process, the extent of the thickness change in the material after rolling can be calculated. This relationship underscores the importance of precise control over the mill parameters to achieve the desired material characteristics of the final product [61,62,63].

Rolling can be performed in various ways, depending on the working temperature and direction, with each method significantly affecting the characteristics of the final product. Hot rolling is conducted above the recrystallization temperature, during which the metal is processed at elevated temperatures to prevent work hardening. At high temperatures, the kinetic energy of the metal atoms increases, making the metal crystal lattice more prone to deformation and allowing for substantial deformation with a lower resistance. In contrast, cold rolling is performed below the recrystallization temperature, typically at room temperature, leading to work hardening. Cold rolling enhances the strength and hardness of the metal, refines the grain structure, and improves the precision and flatness of the sheets. However, at the same reduction rate, the thickness reduction achieved by cold rolling is lower than that achieved by hot rolling. This disparity is attributed to the different atomic movements of the metals at varying temperatures. Cryo-rolling, which is performed at cryogenic temperatures typically below −150 °C, has attracted significant attention for further enhancing the mechanical properties of Ti alloys. This process involves high-temperature heating to initiate deformation, followed by rapid cooling using cryogenic fluids such as liquid nitrogen. Cryo-rolling refines the microstructure, improving both strength and ductility, and is particularly advantageous for high-performance applications, including aerospace and medical implants. Additionally, cryo-rolling promotes the formation of twins while inhibiting dislocation slip, further enhancing the material’s performance.

Additionally, the rolling process can be categorized as unidirectional rolling (UDR), multistep cross-rolling (MSCR), and reverse rolling (RR), depending on the rolling direction [64,65,66]. UDR involves rolling the specimen in the same direction for each pass, which is generally suitable for products that require consistent directional properties. MSCR involves rotating the specimen by 90° in each pass, promoting uniform deformation in various directions, and enhancing the uniformity of the grain structure. In contrast, RR involves rotating the specimen by 180° for each pass, effectively achieving uniform deformation and minimizing the residual stresses. These diverse rolling methods are selectively applied to optimize the physical properties and performance of the final products, establishing them as essential technologies in the metal processing industry.

To better understand the effects of each rolling method on texture and microstructure, a summary of the key advantages and disadvantages is presented in Table 1. This table provides a comparative overview of the impact of hot rolling, cold rolling, cryo-rolling, and various rolling directions (UDR, MSCR, RR) on material properties. By analyzing the advantages and disadvantages of each method, the table emphasizes how specific rolling techniques can be strategically selected based on the desired microstructural and textural outcomes, which is essential for optimizing material performance in various applications.

### 2.2. Texture Analysis

**Figure 3:**
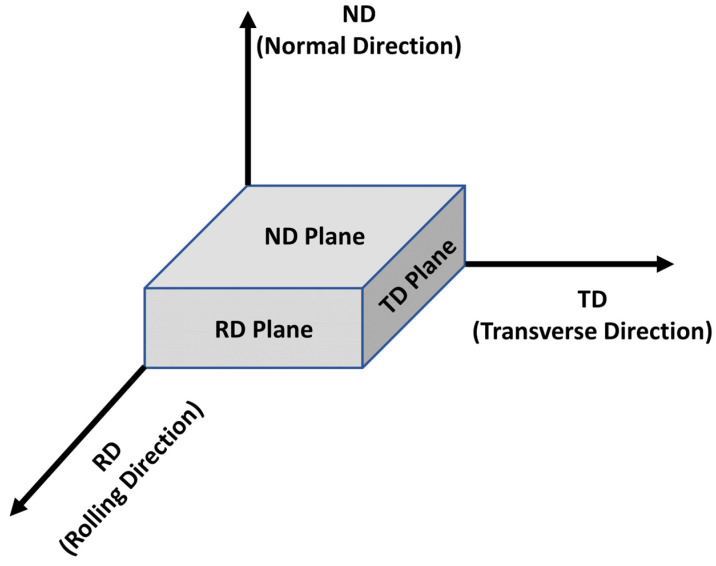
Three-dimensional representation of direction and plane in rolled specimen.

Texture is a component of the microstructure that reflects the directional and regular arrangement of the crystal structure, which significantly affects the mechanical properties and formability of metallic materials. Most metals and alloys consist of grains. The arrangement of these grains in specific directional orientations gives rise to various textures. Texture refers to the state of the microstructure in which the crystallographic orientations of individual grains are aligned in a specific direction rather than randomly. Transverse direction (TD), normal direction (ND), and rolling direction (RD) pertain to directional concepts frequently used in the context of deformation and plastic processing of metallic materials, particularly for describing the arrangement of grains during the rolling process or in microstructural analyses involving texture (Figure 3). Each direction is defined based on the specific deformation direction of the materials, such as metal sheets or blocks. The ND refers to the direction perpendicular to the surface of a metal or alloy. TD signifies the direction perpendicular to both the ND and RD of the metal, whereas RD indicates the direction of the rolling progression. In rolled metals, the RD, ND, and TD are crucial axes related to the arrangement of grains, and the physical and mechanical properties vary depending on these directions. Thus, these concepts are essential for the microstructural analyses of texture. For instance, during the rolling process, as the grains elongate in the RD and are compressed in the ND, differences in the ultimate tensile strength (UTS), YS, and ductility can be observed between the RD and TD.

The texture of Ti significantly influences its mechanical properties, including strength, ductility, and fracture toughness. For instance, a high degree of crystallographic alignment in the texture can lead to increased strength and stiffness in specific directions while potentially reducing the ductility and toughness in others. Various processing techniques such as rolling, forging, and annealing can optimize the texture of Ti alloys for specific applications by controlling the orientation and distribution of grains, thereby achieving customized microstructures and properties that meet specific design requirements [49]. The TD split basal texture is a microstructure observed in Ti after rolling. The basal plane of Ti is the one in which the atoms are the most densely packed and exhibits the highest strength. In the TD split basal texture, the crystallographic basal planes are oriented perpendicular to the TD, whereas the c-axis of the HCP structure remains parallel to the TD. This texture is formed during the deformation processing of the alloy, such as rolling or forging, and is characterized by the splitting of the basal pole figure into two major components separated by 60°. This splitting induces a higher degree of crystallographic alignment along the TD, thereby enhancing the strength and stiffness in that direction.

The texture strength of Ti can be expressed in terms of the multiples of the random distribution (MRD) value, which measures the degree of crystallographic alignment of the material compared with a fully random texture. MRD is calculated as the ratio of crystallographic alignment degree of the measured texture to that of a fully random texture. An MRD value of “one” corresponds to a completely random texture, whereas values greater than “one” indicate a degree of alignment exceeding that expected by chance. For example, an MRD value of five signifies that the strength in a specific crystallographic direction is five times greater than anticipated for a random texture [67,68,69,70,71]. In addition, the concept of random multiples (ROM) is often used interchangeably with MRD. The ROM is essentially the opposite of the MRD, representing the degree to which grains are randomly oriented in multiple directions; a higher ROM value indicates greater randomness without a specific directional alignment. Thus, a higher MRD value suggests a strong texture formation in a specific direction, whereas a higher ROM value indicates a more random grain arrangement. The MRD value can be determined using various techniques such as X-ray diffraction (XRD) or electron backscatter diffraction (EBSD) and serves as a quantitative measure of the texture of Ti and other materials [72].

**Figure 4:**
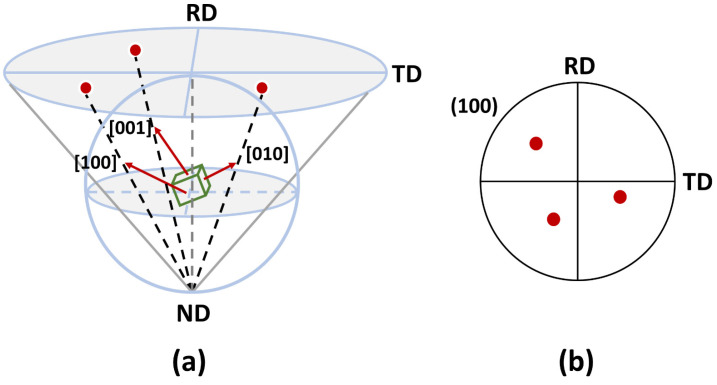
Schematic of pole figure map mechanism: (**a**) projection from sphere to plane, (**b**) two-dimensional projection.

The principle of the pole figure is illustrated in Figure 4, which visually represents the preferred orientation or texture of the crystallographic grains in the analyzed sample [73]. A pole figure is a stereographic projection of the crystallographic directions of the grains onto a two-dimensional circular grid. The circular grid represents all the crystallographic directions of the analyzed sample, with the center of the circle indicating the direction normal to the sample surface and the outer edge representing directions parallel to the surface. In the pole figure, the crystallographic direction of the particles is represented by the poles on the grid, and the size of each pole reflects the proportion of grains oriented in a specific direction. Pole figures can be generated using various techniques such as XRD or EBSD and are extremely useful for understanding the relationship between the crystallographic orientation of grains and the mechanical properties of materials.

### 2.3. Mechanisms of Mechanical Property Improvement

The strengthening mechanisms of metallic materials generally include solid-solution strengthening, precipitation strengthening, dispersion strengthening, work hardening, and grain refinement. Solid-solution strengthening occurs when atoms of different sizes, either interstitially or substitutionally, occupy positions within the metal crystal lattice, causing lattice distortion that enhances the strength of the metal. During precipitation and dispersion strengthening, secondary phases are precipitated within the metal crystal lattice to increase the strength. This is achieved through heat treatments such as solution treatment and aging, which lead to the precipitation of supersaturated solid solutions into secondary-phase particles that hinder the movement of dislocations. Dispersion strengthening involves dispersing compounds, such as oxides, carbides, borides, and nitrides, within the metal matrix to form secondary phases that enhance the strength. In addition, work hardening is achieved by processing the metal to induce plastic deformation, resulting in the generation and increased density of dislocations. Grain refinement reduces the grain size within the metallic material, leading to the formation of a fine microstructure that increases strength. All these methods effectively reduce the slip phenomena within the crystal structure of the metal, thereby enhancing its strength.

Application of plastic deformation through the rolling process leads to grain refinement and work hardening within the material, thereby affecting its properties. Grain refinement enhances the strength of a material due to the slip phenomenon, which corresponds to the movement of dislocations. In polycrystalline materials, slip occurs across grain boundaries during plastic deformation. Grain boundaries obstruct the movement of dislocations, and materials with finer grains have a greater relative area of boundaries that hinder dislocation motion compared with materials with coarser grains, making them harder and stronger [74]. Grain boundaries exhibit higher misorientation angles, which increase the threshold energy required for dislocations to cross grain boundaries, leading to a reduction in slip activity and, consequently, an increase in material strength. Additionally, strain hardening occurs below the recrystallization temperature, manifesting at temperatures lower than the intrinsic melting point of the material. As the degree of deformation increases, the dislocation density within the material rises proportionally, resulting in energy dispersion for slip and enhanced hardness.

(8) $$\sigma_{0}=\sigma_{i}+k^{\prime }D^{-\frac{1}{2}}$$

(9) $$H_{delivery}=\frac{F}{M}+S$$

The increase in strength through grain refinement is represented by the Hall–Petch equation (Equation (8)), where $\sigma_{0}$ is the YS, $\sigma_{i}$ is the slip-resistant frictional stress, k is the dislocation density constant, and D is the grain size. This equation indicates an inverse relationship between grain size and YS [75]. This equation reveals that the average grain diameter significantly influences the mechanical properties of polycrystalline metals. The Taylor equation in Equation (9) represents the relationship between YS, grain size, and dislocation density, where M is the Taylor factor, α is the relative strengthening contribution constant of the grain boundaries, G is the shear modulus, b is the Burgers vector, and ρ is the dislocation density. The Taylor equation illustrates the relationship between the increase in dislocations and the interaction of deformation fields with the mechanical properties [76]. During the work hardening process, as the degree of deformation increases, the dislocation density in the metal also increases, resulting in a decrease in the spacing between dislocations, causing them to be positioned closer together. Consequently, the deformation fields between the dislocations push against each other, and the movement of one dislocation is hindered by the other. Thus, as previously described, an increase in the dislocation density results in greater hindrance to the movement of dislocations, leading to an increase in the YS owing to a reduction in slip.

The difference in the slip systems between BCC and HCP significantly affects their mechanical properties. The HCP structure of alpha Ti has five main slip systems: $(0002)\langle 11\bar{2}0\rangle$ basal <a> slip,$\;\{10\bar{1}0\}\langle 11\bar{2}0\rangle$ prismatic <a> slip, $\{10\bar{1}1\}\langle 11\bar{2}0\rangle$ pyramidal <a> slip, $\{10\bar{1}1\}\langle 11\bar{2}3\rangle$ 1st-order pyramidal <a + c> slip, and $\{11\bar{1}2\}\langle 11\bar{2}3\rangle$ 2nd-order pyramidal <a + c> slip [77]. Although the HCP structure exhibits a relatively high number of slip systems, its asymmetric crystal structure confines them to specific directions and planes. The slip primarily occurs on the {0001} plane and propagates in the $\langle 11\bar{2}0\rangle$ direction. In contrast, the BCC structure of beta Ti possesses three slip systems: $\{100\}\langle 111\rangle$, $\{112\}\langle 111\rangle$, and $\{123\}\langle 111\rangle$ [78,79]. The symmetric BCC crystal structure facilitates slip propagation in multiple directions, with a primary slip direction of $\langle 111\rangle$. Owing to these differences in the crystal structures, BCC Ti allows easier dislocation movement, resulting in superior ductility and formability, whereas HCP Ti generally exhibits lower ductility owing to restrictions in the slip directions and planes. Therefore, the operational slip systems arising from the differences between HCP and BCC structures are critical factors in the changes in mechanical properties.

Twinning is a form of deformation that occurs in metallic materials when they are subjected to strain or stress. It forms small regions within the grains, where the twinning areas exhibit a directional nature and are symmetrically arranged along specific boundary planes. This twinning effect leads to rearrangement of the crystal structure, resulting in the formation of defects or hardened structures within the grains, thereby affecting the mechanical properties of the metal. For Ti, tensile twinning primarily occurs in $\{11\bar{2}1\}\langle 11\bar{2}\bar{6}\rangle$ and $\{10\bar{1}2\}\langle 10\bar{1}\bar{1}\rangle$ types, whereas compressive twinning is observed in $\{11\bar{2}2\}\langle 11\bar{2}\bar{3}\rangle$, $\{11\bar{2}4\}\langle 22\bar{4}\bar{3}\rangle$, and $\{10\bar{1}1\}\langle 10\bar{1}\bar{2}\rangle$. HCP metals typically possess relatively few slip systems, which necessitate twinning during plastic deformation [80,81]. Therefore, twinning plays a crucial role in the plastic deformation of Ti, making it essential to select twinning as the appropriate deformation mechanism that influences the development of texture and microstructure.

## 3. Various Rolling Methods for Ti and Its Alloys

### 3.1. Classification of Rolling Processes by Temperature

#### 3.1.1. Cold-Rolling Process

Cold rolling is a process in which materials are rolled at room temperature and is typically performed below the recrystallization temperature. This process induces significant internal stresses, leading to work hardening. Work hardening occurs when a material is subjected to stresses beyond its YS, resulting in a permanent deformation that prevents the material from returning to its original shape even after the external force is removed. This form of plastic deformation increases the density of the microstructure, thereby enhancing its resistance to slip without causing material failure. Consequently, in cold rolling, work hardening increases the grain boundary density within the microstructure, impeding slip and resulting in higher strength. However, as cold rolling is performed at low temperatures, the reduction rate is low. To compensate for this, the process is conducted at temperatures approximately half that of the recrystallization temperature, which is higher than room temperature. Consequently, various studies have been conducted to achieve increased strength through significant microstructural changes.

Liu et al. studied the effects of cold rolling and heat treatment on the microstructure and texture of CP-Ti sheets [82]. The microstructure and texture of CP-Ti during cold rolling and heat treatment were analyzed using as-received CP-Ti with dimensions of 230 mm (length) × 60 mm (width) × 4.6 mm (height) and a composition of Ti-0.02Fe-0.039O-0.003N (wt%). Cold rolling was performed at reduction rates of 10%, 20%, 30%, and 40% to examine the microstructure and texture changes during plastic deformation. To investigate the microstructural changes in CP-Ti during heat treatment, the specimens with a 20% reduction rate were subjected to heat treatment for 5, 10, 20, 30, and 60 min. Following heat treatment, the specimens underwent electrolytic polishing, and microstructure and texture analyses were conducted using EBSD.

**Figure 5:**
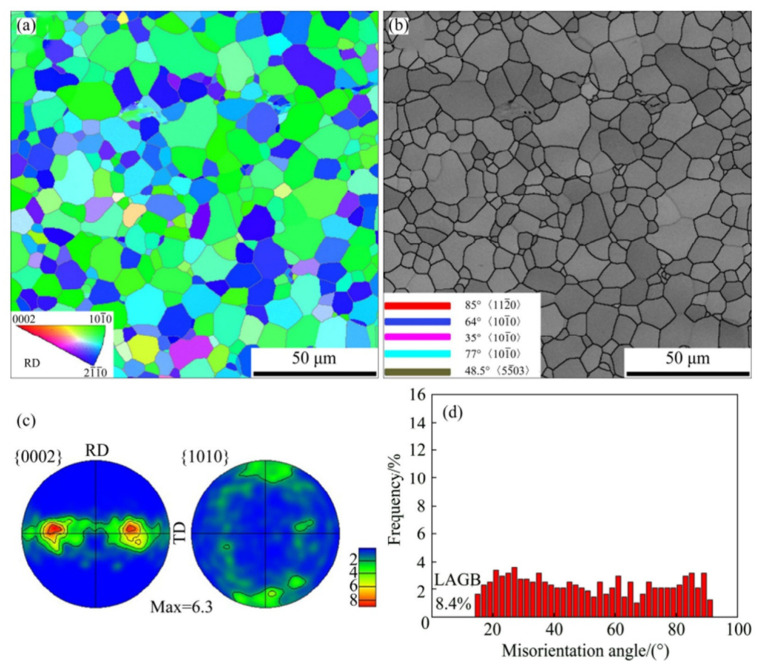
EBSD data analysis of as-received CP-Ti: (**a**) inverse pole figure (IPF) map, (**b**) image quality (IQ) map, (**c**) pole figure, and (**d**) misorientation angle distribution [82].

Figure 5 shows the EBSD data of the initial CP-Ti (as received) before the rolling process. In Figure 5a, the inverse pole figure (IPF) color map indicates that the average grain size is 10 μm. In Figure 5b, the image quality (IQ) map reveals that twinning does not occur in the initial grains where no plastic deformation has occurred. Pole figure analysis in Figure 5c indicates that most grains are dispersed along the TD based on the (0002) plane, which aligns with the C axis in the ND. The misorientation angle distribution analysis in Figure 5d divides the boundaries into high-angle grain boundaries (HAGBs) and low-angle grain boundaries (LAGBs) based on a 15° threshold. The initial CP-Ti consisted of 8.4% LAGBs and 91.6% HAGBs. Therefore, the initial (as-received) sample possessed a uniform microstructure consisting predominantly of HAGBs.

**Figure 6:**
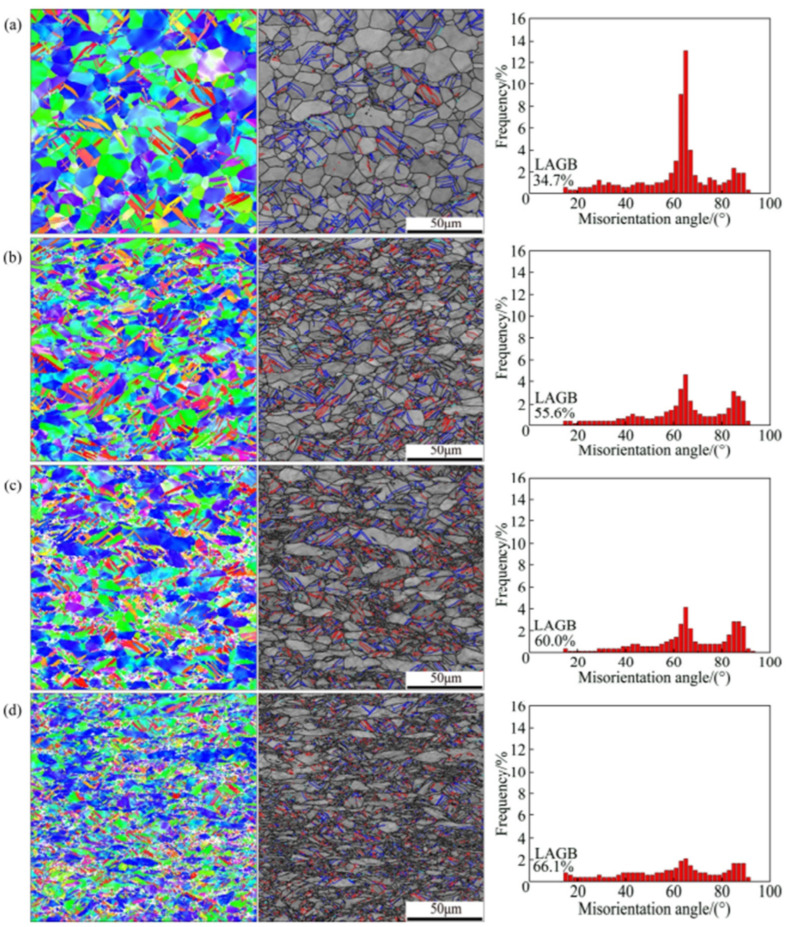
EBSD IPF maps, IQ maps, and misorientation angle distributions after cold-rolled CP-Ti samples with different reduction rates of (**a**) 10%, (**b**) 20%, (**c**) 30%, and (**d**) 40% [82].

When CP-Ti undergoes a rolling process with a reduction rate of 10–40%, this study identifies twinning as a primary mechanism of plastic deformation, which facilitates grain refinement and influences the evolution of texture and mechanical properties. Specifically, $\left\{10\bar{1}2\right\}$ extension twins and $\left\{11\bar{2}2\right\}$ contraction twins are activated depending on the stress state and reduction rate. The IPF and IQ maps of CP-Ti in Figure 6a (10% reduction) reveal the occurrence of twinning within the material. The IQ map distinguishes between two types of twins: $\left\{11\bar{2}2\right\}\langle 11\bar{2}\bar{3}\rangle$ contraction twins observed at a misorientation angle of 60–65° and $\left\{10\bar{1}2\right\}\langle 10\bar{1}1\rangle$ extension twins observed at a misorientation angle of 80°. Changes in twinning can be confirmed through the IQ map and misorientation angle distribution graphs; for a 10% reduction, the proportion of contraction twins at 60–65° is higher than that of extension twins at 80°. In Figure 6b, the results for a 20% reduction show that compared to a 10% reduction, the activation of extension twins at 80° is relatively greater.

**Figure 7:**
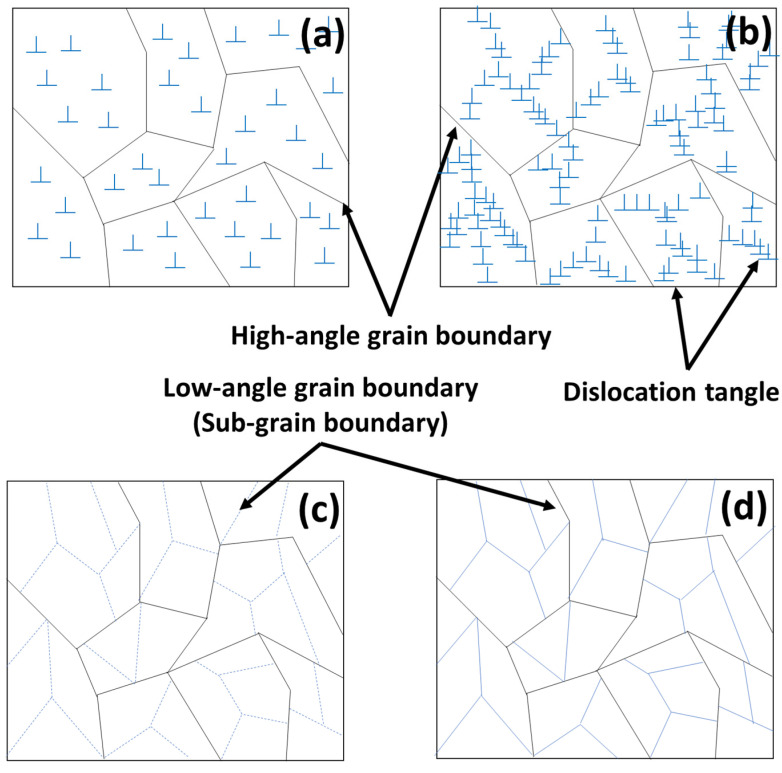
Schematics for formation of low-angle grain boundary (LAGB) (sub-grain boundary). (**a**) Before cold rolling, (**b**) after cold rolling, and (**c**,**d**) formation of LAGB (subgrain boundary).

This study further highlights that the interaction between dislocations and twins leads to the formation of subgrains, as evidenced by the increased proportion of low-angle grain boundaries (LAGBs), a hallmark of work hardening during cold rolling. In the IQ maps for reductions of 30% and 40% shown in Figure 6c,d, most grains exhibit elongation in the RD, indicating significant grain refinement. This grain refinement is attributed to twinning, which intensifies as the strain increases. Twinning facilitates grain boundary rearrangements, introducing geometric constraints that inhibit grain growth, as observed under similar rolling conditions. The rearrangement of atomic structures due to twinning results in an increased dislocation density at the boundaries, further promoting grain refinement. Research has demonstrated that the interaction between twins and dislocations generates subgrains during plastic deformation, which evolve into low-angle grain boundaries (LAGBs) under cold working conditions. These subgrains play a significant role in refining the microstructure and enhancing mechanical properties. Additionally, the overall increase in reduction correlates with an increase in the proportion of LAGBs, which, as shown in Figure 7, is attributed to the formation of subgrains owing to cold rolling. Tangled dislocations arise from the increased dislocation density during cold rolling, and during the recovery process after cold working, tangled dislocations transform into LAGBs.

**Figure 8:**
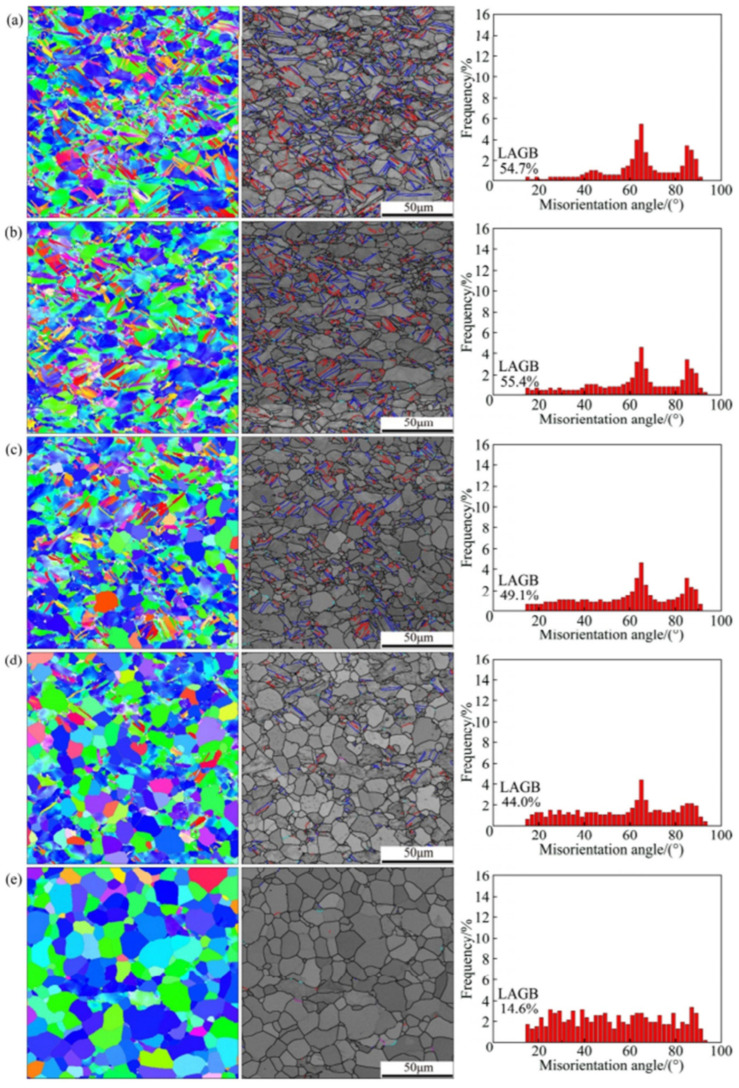
EBSD IPF maps, IQ maps, and misorientation angle distributions for 20% reduction sample after annealing at 550 °C with various times: (**a**) 5 min, (**b**) 10 min, (**c**) 20 min, (**d**) 30 min, and (**e**) 60 min [82].

Heat treatment following cold rolling has a significant impact on the microstructure and mechanical properties of CP-Ti. During rolling, the increased dislocation density and twin formation enhance strength but also introduce residual stresses. Heat treatment at 550 °C alleviates these stresses by promoting dislocation recovery and grain boundary migration. Figure 8 shows the effects of heat treatment on the microstructure over various times. When heat treatment is performed at 550 °C for 5 min, no change in the grain shape or the number of twins is observed. However, when the heat treatment is extended beyond 10 min, the proportion of twins decreases and the grain size increases. The results of a 60 min heat treatment show that the microstructure returns to its pre-processing state owing to recrystallization. Additionally, the misorientation angle distribution reveals that the peaks at approximately 65° and 85° gradually decrease as the heat treatment time increases. This is because the number of contraction twins corresponding to the {$11\bar{2}2\}$ plane and extension twins corresponding to the {$10\bar{1}2$} plane decreases during heat treatment. With an increase in the heat treatment time, the proportion of LAGBs gradually decreases, whereas the proportion of HAGBs decreases sharply, indicating that the consumption of twins during grain recrystallization is more pronounced during heat treatment. Consequently, the density of dislocations acting as internal stress and the proportion of twins decrease through the heat treatment of the work-hardened metal, leading to the growth of new grains, an increase in the grain size, and an increase in the proportion of HAGBs. This observation of microstructural changes during heat treatment further emphasizes the importance of understanding how rolling and heat treatment together influence both the microstructure and mechanical properties of CP-Ti. The results from the EBSD analysis during rolling and the subsequent effects of heat treatment highlight the dynamic interplay between dislocation density, twinning, and grain refinement. Such detailed analysis can contribute to optimizing processing techniques for improved material properties.

**Figure 9:**
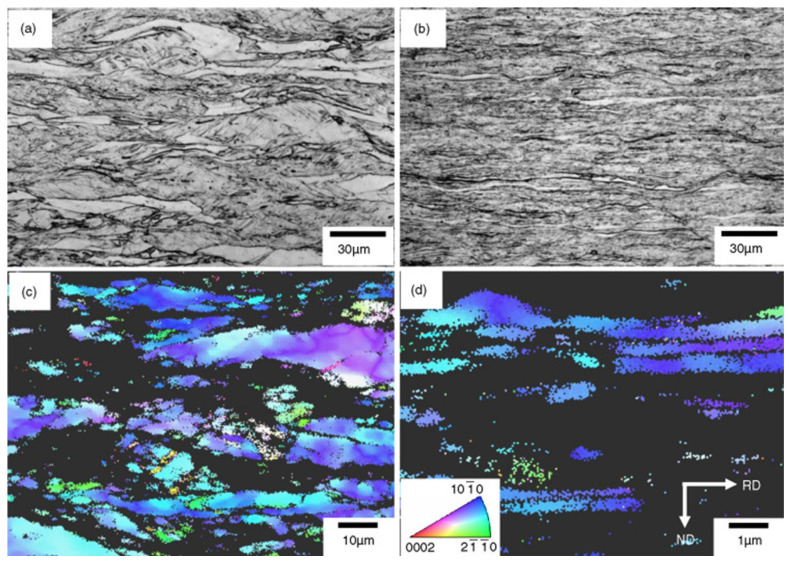
Microstructures of CP-Ti samples after cold rolling with different reduction degrees: (**a**) 60% (optical micrograph), (**b**) 90% (optical micrograph), (**c**) 60% (EBSD-orientation image), and (**d**) 90% (EBSD-orientation image) [83].

Hwang et al. conducted a microstructure and texture analysis of CP-Ti subjected to cold rolling, with a significant reduction of over 40% [83]. Figure 9 depicts the microstructure of Ti, illustrating that Ti, subjected to a 60% reduction, has coarse grains approximately 10 μm in size that are elongated in the RD, along with finer grains due to twinning. The average particle size of the initial CP-Ti was 30 μm in an equiaxed form, indicating that the increase in aspect ratio represents the changes associated with the high reduction following the 60% rolling process. In the EBSD analysis represented in Figure 9c, a directional orientation is observed in the range of $\varphi_{1}$ = 0°, *φ* = 30–90°, and $\varphi_{2}$ = 30°, indicating data loss associated with high reductions. Furthermore, in Figure 9b, corresponding to a 90% reduction, the microstructure demonstrates refinement and macroscopic shear bending due to the high reduction, with the size of elongated grains decreasing to 3 μm. The directional image of the sample rolled with a 90% reduction, shown in Figure 9d, reveals that the lattice has been excessively deformed, resulting in the inability to analyze 70% of the data points, with a directional orientation in the range of $\varphi_{1}$ = 0°, *φ* = 30–50°, and $\varphi_{2}$ = 30°. This indicates that, as the amount of deformation increases, the primary pole near the TD shifts toward the ND.

**Figure 10:**
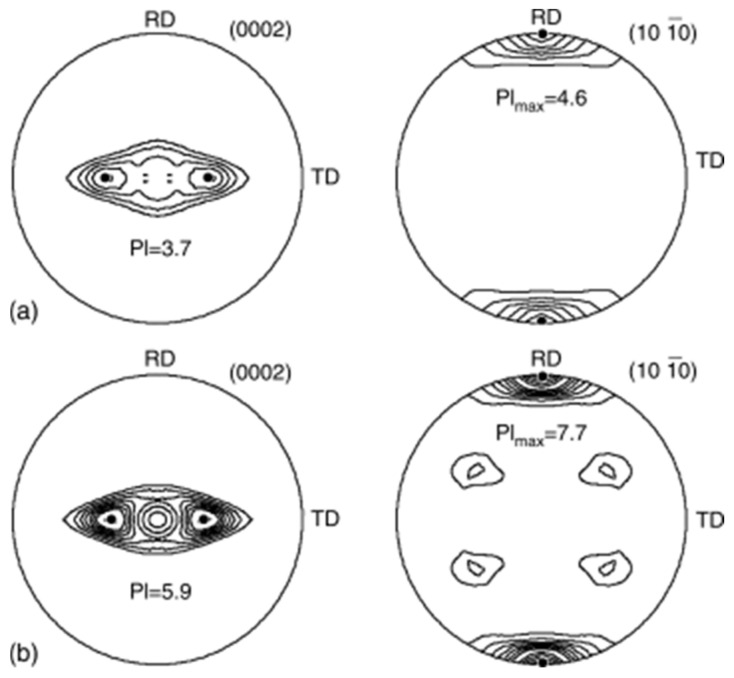
Pole figure map of CP-Ti after cold rolling with (**a**) 60% and (**b**) 90% reduction [83].

The split basal texture reappears at higher levels of deformation, as illustrated in Figure 10. After a 60% reduction, the basal pole exhibits an MRD value of 3.7, while after a 90% reduction, the split basal texture is found to be tilted ±35° from the ND toward the TD, with an MRD value of 5.9. For the CP-Ti reduced by 40%, the (0002) pole figure is observed. However, upon a 60% reduction, the (1010) pole figure of the rolled Ti shows an MRD value of 4.6 in the RD. Consequently, by increasing the reduction, the strength of the prism poles is enhanced, reaching an MRD value of 7.7. This phenomenon demonstrates how cold rolling affects the fundamental texture of Ti; at reduction rates of 10% to 40%, the strength of the split basal planes gradually increases in the ND. However, at higher deformation levels (>60%), the texture splits toward the TD, resulting in a subsequent increase in the MRD value.

**Figure 11:**
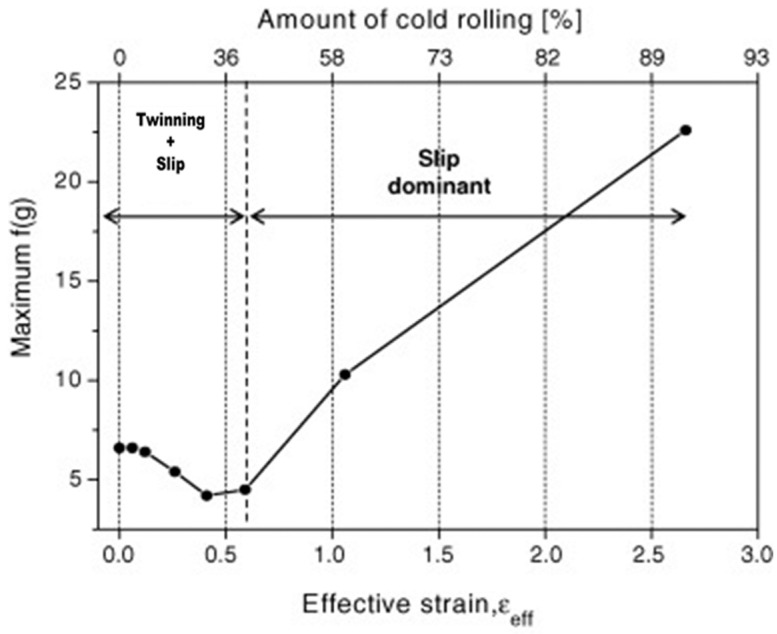
Variation of the maximum intensity of f(g), indicating the effects of twinning and slip activation in weakening in cold-rolled CP-Ti [83].

As shown in Figure 11, cold reductions below 40% lead to the activation of twinning and slip. At lower reduction rates, twinning becomes more dominant, as relatively less deformation occurs, promoting its activation within the grains. This leads to a lower maximum f(g) value, which represents the distribution density of crystallographic directions (g) while maintaining a balance between the two mechanisms. However, when the reduction exceeds 40%, the increased deformation limits the activation of twinning and shifts the primary deformation mechanism to dislocation motion. As a result, dislocations align along the deformation direction, leading to an increase in the maximum f(g), indicating stronger crystallographic orientation. Understanding this transition from twinning to dislocation motion is crucial for comprehending the evolution of deformation mechanisms during cold rolling. By examining the changes in texture strength with increasing reduction, the influence of each mechanism, such as twinning and slip, can be clearly identified, providing valuable insights into the material’s response to plastic deformation.

**Figure 12:**
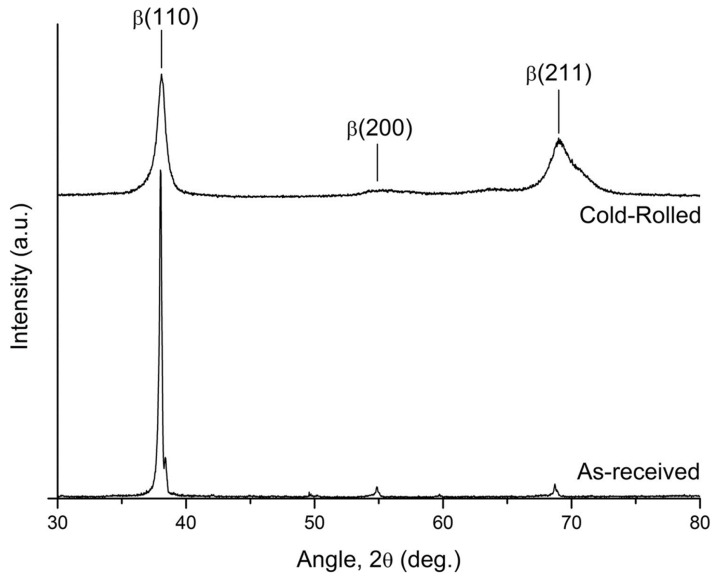
XRD patterns of as-received and cold-rolled Ti-25Nb-25Zr samples [84].

Vajpai et al. studied the microstructure and mechanical properties of Ti-25Nb-25Zr (wt%), which has a β-type cubic structure instead of an α-Ti HCP structure, through cold working [84]. Figure 12 shows the XRD patterns of the cold-rolled Ti-25Nb-25Zr alloy specimens, along with those of the initial alloy that underwent solution treatment for comparison. The cold-rolled specimens exhibited only the β phase, consistent with the solution-treated specimens, indicating that cold rolling did not induce any phase transformation. However, the diffraction peaks of the cold-rolled specimens were noticeably broader than those of the solution-treated specimens. Furthermore, the relative intensities of the cold-rolled specimens were significantly reduced compared to those of the solution-treated specimens. These changes might be attributed to grain refinement caused by cold rolling, which reduces grain size. The observed peak broadening and intensity reduction indicate that the plastic deformation introduced during cold rolling leads to a finer grain structure and significant microstructural changes in the alloy.

**Figure 13:**
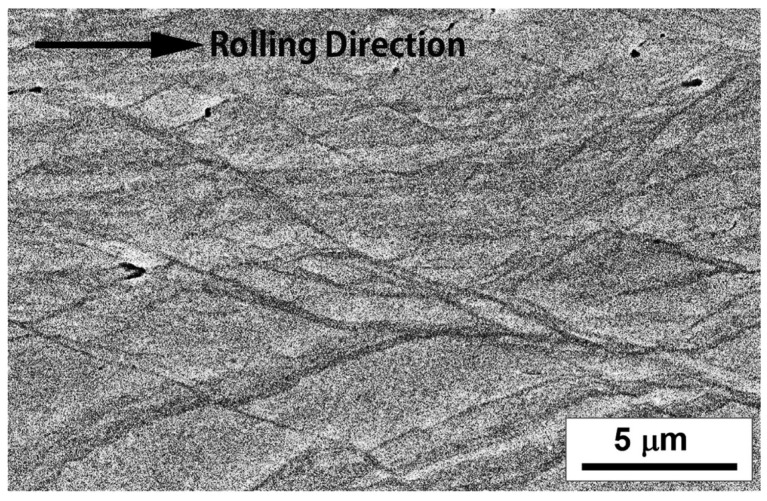
Microstructure of cold-rolled Ti-25Nb-25Zr specimens after cold rolling [84].

Figure 13 shows a representative microstructure observed in a thickness-direction cross-section of cold-rolled Ti-25Nb-25Zr specimens with 80% reduction. This microstructure clearly exhibits severely deformed, elongated, and subdivided grains, along with numerous shear bands. These microstructural characteristics indicate that the specimens experienced a significant level of plastic deformation during the cold-rolling process. Unlike high-temperature deformation, cold rolling is performed at low temperatures where recrystallization does not occur, indicating that the deformation within the microstructure is primarily manifested through dislocation tangles, grain refinement, and the formation of shear bands. The observed shear bands are a result of the strong localized shear deformation that occurs during cold deformation, indicating that the deformed grains are predominantly elongated and fragmented, leading to highly abnormal shapes. These shear bands are one of the main deformation mechanisms observed in cold-rolled metals and can significantly alter the mechanical properties of the material. The microstructural changes identified in the cold-rolled Ti-25Nb-25Zr specimens suggest that substantial microstructural alterations can occur owing to the accumulation of plastic deformation, even in the absence of phase transformation, indicating the important role of the rolling process in controlling the mechanical properties of the alloy.

**Figure 14:**
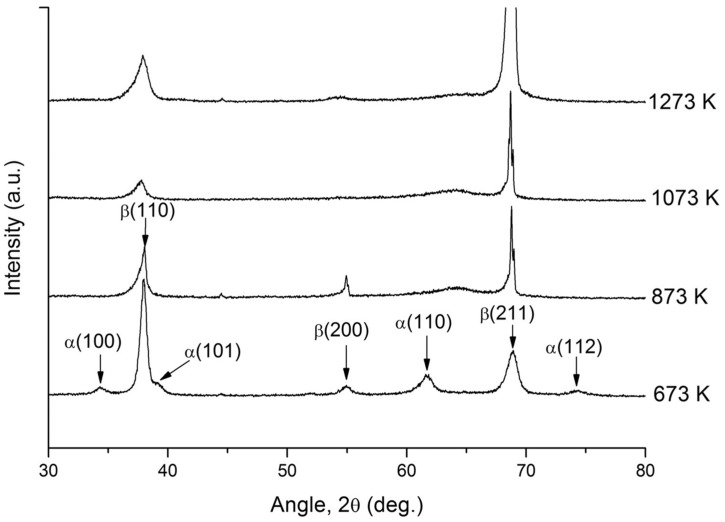
XRD patterns of cold-rolled Ti-25Nb-25Zr samples after annealing at 673 K, 873 K, 1073 K, and 1273 K [84].

Figure 14 shows the XRD patterns of the Ti-25Nb-25Zr alloy after heat treatment at temperatures ranging from 673 to 1273 K. The results show that the β phase is predominantly present in all specimens, with the α phase (HCP) being observed only in the specimen treated at 673 K in small amounts. Additionally, the relative intensity of the XRD peak corresponding to the (211) plane of the β phase increases proportionally with the increase in temperature, indicating that heat treatment enhances the crystallographic orientation. Consequently, it can be concluded that low-temperature annealing at 673 K induces relaxation and recovery effects of the deformation energy rather than a recrystallization effect, while simultaneously leading to the precipitation of the α phase (HCP). The findings presented in this study indicate that heat treatment following the cold-rolling process can significantly influence the microstructural evolution of the Ti-25Nb-25Zr alloy. Cold rolling introduces significant plastic deformation, leading to grain refinement, while subsequent heat treatment at various temperatures further alters the crystallographic orientation and phase stability. This process not only facilitates the recovery of deformation-induced energy but also promotes the precipitation of the α phase at lower temperatures. The synergistic effect of cold rolling and heat treatment is critical for optimizing key properties of the alloy, including strength and phase distribution, thereby improving its applicability for specific functional requirements.

#### 3.1.2. Hot-Rolling Process

In general, hot rolling involves heating a material to a high temperature and performing rolling above the recrystallization temperature. This process allows the application of high reduction rates without causing strain hardening in the metal, thus enabling increased deformation and reduced processing time. During hot rolling, the surface of the material reacts with oxygen at high temperatures, leading to oxidation and resulting in a relatively rough and detached surface. However, in the case of Ti alloys, hot rolling above a certain temperature can cause the precipitation of specific phases and the recrystallization of particles, which in turn affects the strength through changes in the microstructure. Studies have demonstrated the influence of achieving a uniform microstructure and the properties of specific microstructures on the strength of Ti alloys.

**Figure 15:**
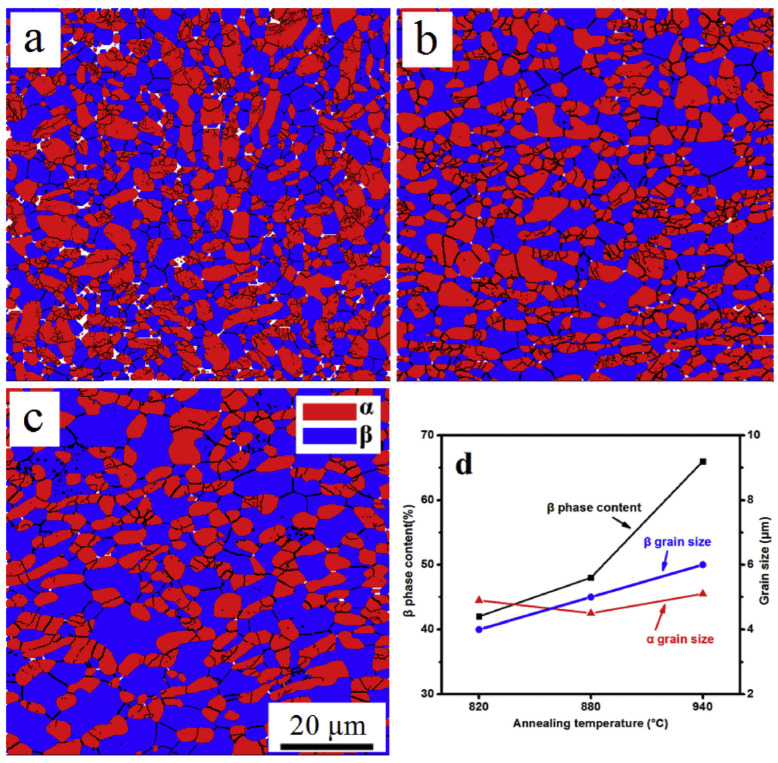
Phase map after annealing at (**a**) 820 °C, (**b**) 880 °C, and (**c**) 940 °C of TC21 Ti alloy. (**d**) Content of β phase in relation to annealing temperature and grain size [85].

Wang et al. conducted a hot-rolling study using a TC21 Ti alloy rod with a chemical composition of Ti-6.28Al-3.06Mo-1.89Nb-2.04Sn-2.18Zr-1.61Cr-0.066Si-0.022Fe-0.009C-0.122O-0.005N (wt%), which was hot-forged with a diameter of 300 mm to create a uniform microstructure through precipitation and recrystallization [85]. Prior to the hot rolling, three specimens were subjected to an annealing step at 860 °C for 1 h. Then, hot rolling was performed at a temperature of 750 °C with a thickness reduction rate of 15% per pass, leading to a total thickness reduction rate of 60%. Following this, the three specimens underwent an annealing process for 1 h at temperatures of 820 °C, 880 °C, and 940 °C, respectively, before being analyzed. Figure 15 shows the ratio of β phase and the particle size differences between α phase and β phase as a function of the annealing temperature. The ratio of the β phase increases with the annealing temperature, approaching 960 °C, which is the temperature at which all phases of this alloy transform into β phase.

This study investigated the control of processing conditions to achieve uniform α phase and β phase by examining dynamic recrystallization and recovery occurring during hot rolling, as well as static recrystallization occurring during annealing. During hot rolling, the α phase has a low critical shear stress, which activates prism slip significantly, resulting in efficient recrystallization and recovery, leading to an elongated shape. In contrast, the β phase experiences minimal recrystallization and recovery despite enduring high deformation. After hot rolling, the α phase and β phase exhibit a non-uniform microstructure. Thus, efforts have been made to obtain uniform microstructures via high-temperature annealing.

**Figure 16:**
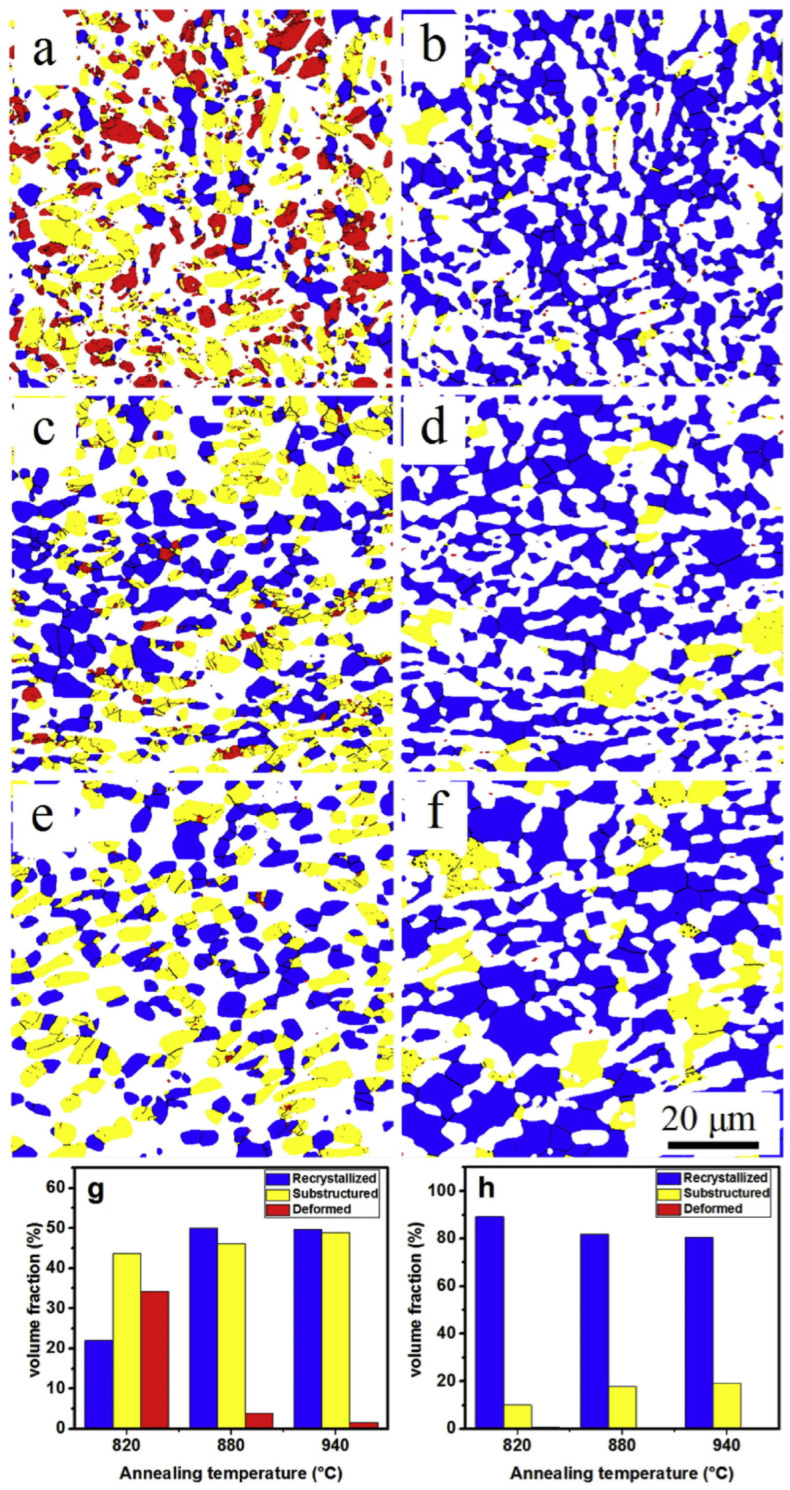
Recrystallization, substructured and deformed α (**a**,**c**,**e**) phase, β (**b**,**d**,**f**) phase, and volume fraction (**g**,**h**) after annealing process are presented by the internal average misorientation angle (IAMA) map. At 820 °C (**a**,**b**), 880 °C (**c**,**d**), and 940 °C (**e**,**f**) annealing temperatures. Effect of volume fraction on (**g**) α phase and (**h**) β phase [85].

**Figure 17:**
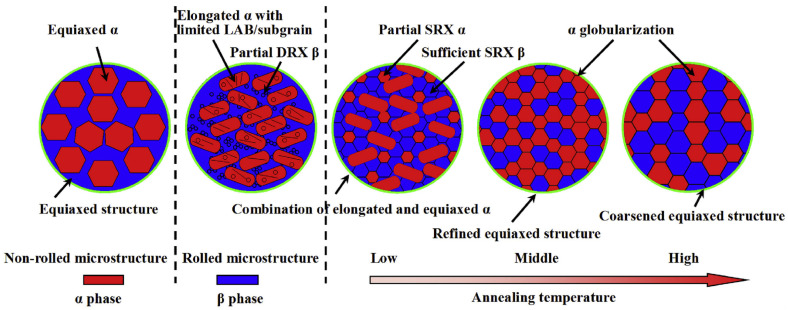
Schematic showing the recrystallization and grain growth during hot rolling and annealing (at 840 °C, 880 °C, and 960 °C, respectively) [85].

Figure 16 illustrates recrystallization, deformation, and intermediate structures at each annealing temperature. As the annealing temperature increases, the proportion of β phase particles also increases, leading to significant static recrystallization and growth of β phase, resulting in a uniform microstructure with α phase. Among the three annealing temperatures, annealing at 880 °C produces a very uniform microstructure, whereas annealing at 960 °C causes the particles to grow larger, resulting in a non-uniform microstructure. Figure 17 illustrates the phase transformations of α and β phases during the various stages of processing, including hot rolling and subsequent annealing. The microstructural evolution is closely related to the deformation mechanisms and static recrystallization (SRX) behavior that occur during these processes. Before hot rolling, the microstructure primarily comprises equiaxed α and β phases. During rolling, the β phase undergoes slight dynamic recrystallization (DRX) due to its higher deformation capacity, while the equiaxed α grains become elongated, with minimal formation of low-angle grain boundaries (LAGBs) and subgrains. Consequently, SRX occurs more effectively in β phase, resulting in a transition toward an equiaxed morphology. With annealing, the grain size progressively increases as subgrains and recrystallized grains grow, particularly in β phase. The annealing process, in conjunction with hot rolling, is instrumental in refining and homogenizing the grain structure, producing a more uniform microstructure. As the annealing temperature rises, the β phase content increases, enhancing SRX activity and further promoting microstructural uniformity. This combined strategy effectively leverages the interplay between hot rolling and heat treatment to tailor the material properties of the TC21 Ti alloy. The resulting uniform microstructure is advantageous for high-performance applications. This analysis underscores the critical role of optimizing both hot-rolling and annealing processes to achieve enhanced material performance. The observed transformations emphasize the importance of controlling these processing steps to fine-tune grain structure and phase distribution, ensuring the desired mechanical properties.

Su et al. analyzed the effects of microstructural differences in Ti alloys, based on the hot-rolling temperature, on their strength [86]. The study utilized a DsTi700 alloy with a chemical composition of Ti-6.02Al-2.70Sn-9.83Zr-0.76Mo-0.91Nb-0.98W-0.29Si (wt%) in dimensions of 70 × 60 × 15 mm^3^. Prior to hot rolling, the specimens were ground and coated to prevent oxidation. Hot rolling was conducted over a total of six passes at temperatures of 1000 °C, 1025 °C, 1050 °C, and 1100 °C. The first three passes achieved a thickness reduction of 60%, whereas the final three passes further reduced the thickness to approximately 3 mm, resulting in a total thickness reduction of 50% relative to that of the original rolled material.

**Figure 18:**
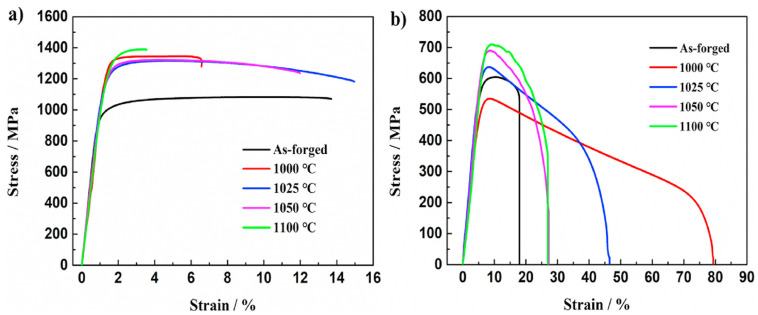
Results of tensile stress curve at (**a**) room temperature and (**b**) 650 °C [86].

As shown in Figure 18, as the rolling temperature increases, both the YS and UTS initially decrease before increasing, whereas the elongation first increases to a maximum before sharply decreasing. The highest UTS value recorded was 1390 MPa at 1100 °C, while lower UTS and YS were observed at 1025 °C and 1050 °C, despite a predominant elongation of over 10%a. Ti alloys with equiaxed or dual-phase microstructures typically exhibit high ductility. However, at 1000 °C, low ductility is observed despite these microstructural characteristics. This can be attributed to the abnormal phenomena caused by the condensation of S2 (silicides) within the matrix, which leads to stress concentration and a reduction in ductility.

**Figure 19:**
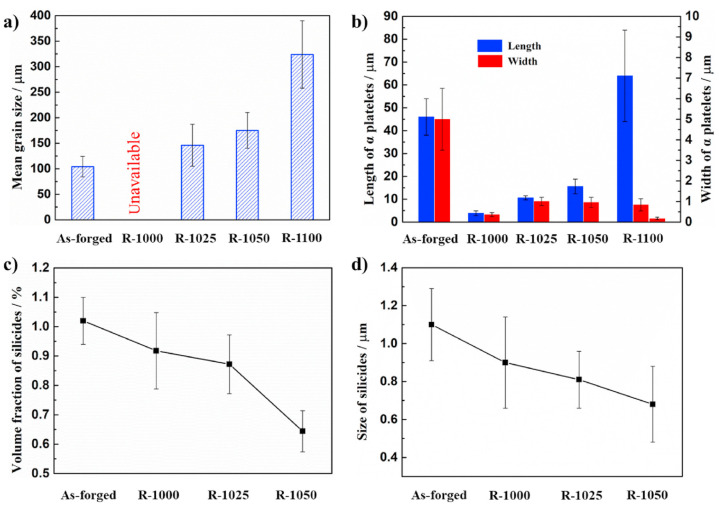
Microstructure behavior with rolling temperature: (**a**) mean grain size, (**b**) length and width of α platelets, (**c**) volume fraction of S_2_ silicides, and (**d**) size of S_2_ silicides [86].

As shown in Figure 19, the size of β particles increases with rising temperature, particularly when the rolling temperature exceeds 1025 °C. At 1100 °C, the particle size is nearly twice as large compared to the sizes observed at 1025 °C and 1050 °C. In contrast, α platelets undergo refinement after rolling, exhibiting a narrow and thin width. At 1100 °C, silicides are not detected. This can be explained by the increased solid solubility of the alloy containing Zr and Si with temperature, leading to their dissolution at higher rolling temperatures and complete disappearance at 1100 °C. Dislocations primarily occur in the long and thin α phase during the rolling process as they rearrange to occupy positions between the particle boundaries. The high self-diffusion of β phases facilitates dislocation movement, resulting in the homogenization of the dislocation substructure. Comparing 1025 °C and 1050 °C, rolling at 1100 °C reduces dislocation activity due to atomic thermal activation, while longer recovery times lead to a more pronounced elimination of dislocations.

**Figure 20:**
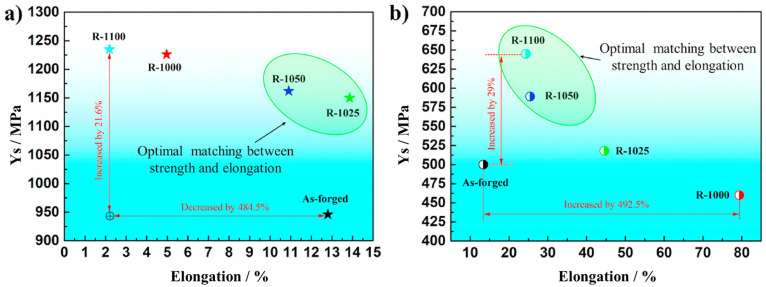
Properties of tensile strength at (**a**) room temperature and (**b**) 650 °C [86].

Figure 20 clearly demonstrates an improvement in the tensile strength at room temperature, with all the rolled sheets exhibiting YS above 1150 MPa. However, at 650 °C, a more significant enhancement in ductility is observed, while strength improvements are observed only at higher rolling temperatures. In conclusion, as the rolling temperature increases from 1000 °C, the proportion and size of S2 silicides gradually decrease, with their complete disappearance confirmed at 1100 °C. For this reason, the high strength values at 1100 °C are associated with lower ductility, while at 1000 °C, the presence of an equiaxed and dual microstructure is compromised by the condensation of S2 silicides within the matrix, resulting in low ductility. It was concluded that the intermediate temperatures of 1025 °C and 1050 °C exhibit an optimal relationship between strength and ductility.

Zhu et al. investigated the effect of the phase transformation and recrystallization processes during hot rolling on the microstructure and mechanical properties of Ti alloys [87]. This research was conducted using a Ti alloy with a chemical composition of Ti-5.1Al-2.5Cr-0.5Fe-4.5Mo-1.1Sn-1.8Zr-2.9Zn (wt%) that underwent hot rolling at 970 °C. After nine passes, the final thickness was 66.7% of the initial thickness.

**Figure 21:**
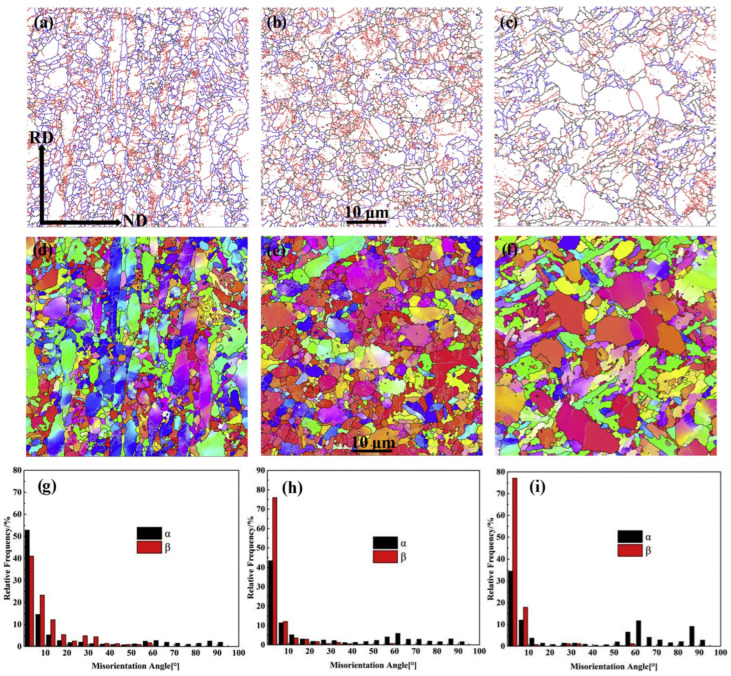
EBSD analysis results of hot-rolled Ti alloy sheet: (**a**–**c**) GB (grain boundary) maps, (**d**–**f**) IPF color grain boundaries, (**g**–**i**) misorientation distribution maps. (**a**,**d**,**g**): S = 0; (**b**,**e**,**h**): S = 0.4; (**c**,**f**,**i**): S = 0.8 [87].

To observe the microstructure, the rolled specimens were divided into three sections: S = 0 (center), S = 0.4 (2/5 position), and S = 0.8 (4/5 position). As shown in Figure 21, the average particle size at S = 0 was 1.68 μm, with α and β phase ratios of 65.6% and 34.4%, respectively. At S = 0.4, the average particle size increased to 1.88 μm, with α and β phase ratios of 86% and 14%. At S = 0.8, the average particle size further increased to 1.98 μm, with α and β phase ratios of 65.6% and 34.4% in Figure 21a, 86% and 14% in Figure 21b, and 92.4% and 7.6% in Figure 21c. This indicates that the proportion of α phase increases as one moves away from the midpoint, suggesting a correlation of temperature distribution with thickness.

In Figure 21a–c, LAGBs (less than 15°) are shown in red, HAGBs (greater than 15°) are displayed in black, and phase boundaries are shown in blue. At S = 0, the percentage of HAGBs in the β phase was 23.4%, while in the α phase, it was 27.2%. This suggests that low-angle boundaries in the β phase are transitioning to high-angle boundaries, primarily due to recovery in the β phase and dynamic recrystallization in the α phase. At S = 0.4, the percentage of HAGBs in the β phase decreased to 8.4%, while it increased to 40% in the α phase, indicating greater dynamic recrystallization in the α phase compared to S = 0.

At S = 0.8, the percentage of HAGBs in the β phase was 3.9%, while in the α phase, it reached 49.9%. Although dynamic recrystallization was more prevalent in the α phase, the degree of recrystallization in the β phase was the lowest. The increasing dynamic recrystallization in the α phase as one moves from the center to the surface of the rolled material may be related to the distribution of accumulated deformation energy and varying temperatures during the hot rolling. The transformation from α to β phase occurs at the highest temperatures in the middle section, primarily driven by weak recrystallization of the α phase and cooling, predominantly resulting from deformation energy and heat. As the distance from the midpoint increases, both deformation energy and temperature decrease, which inhibits the transformation from α to β phase and increases the driving force for recrystallization of the α phase.

**Figure 22:**
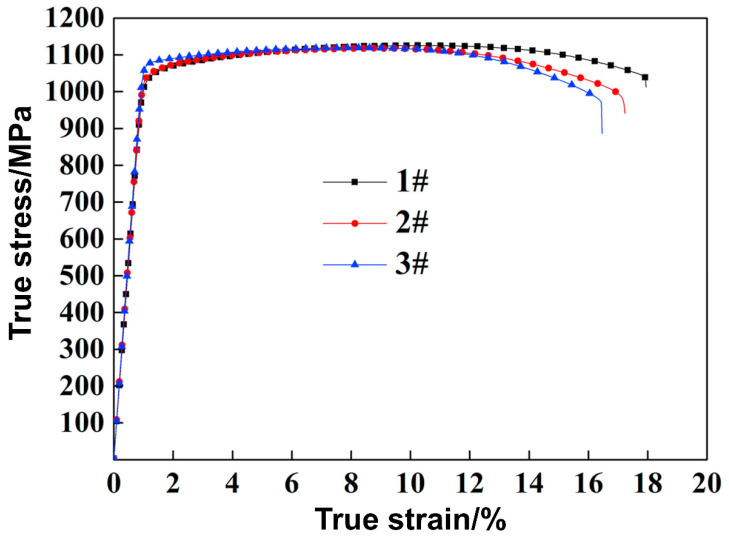
True stress–strain curves with thickness of specimen. #1: S = 0, #2: S = 0.4, #3: S = 0.8 [87].

As shown in Figure 22, Specimen #1 exhibited the lowest YS of 1035 MPa along with the highest elongation of 17.9%. In contrast, Specimen #3 had the highest YS of 1075 MPa but the lowest elongation of 16.4%. Specimen #2 showed a YS of 1043 MPa and an elongation of 17.1%. Moving away from the midpoint, the YS and elongation displayed opposite trends. The factors influencing strength and ductility were found to vary according to the ratio of α and β phases. The strength in the BCC (β phase) is higher than that in the HCP (α phase). Consequently, an increase in the proportion of α phase naturally leads to a decrease in strength. In conclusion, the relatively smaller average particle size at the midpoint, combined with lower recrystallization of the α phase and a higher presence of the BCC (β phase), contributes to the relatively high strength observed in this specimen.

#### 3.1.3. Cryogenic Rolling Process

Cryo-rolling was utilized to enhance the mechanical properties of Ti and its alloys. This operation involves exposing the material to cryogenic temperatures, typically below −150 °C (−238 °F), in conjunction with rolling or other deformation processes. During the cryo-rolling process, Ti alloys are initially heated to high temperatures to facilitate deformation. After that, they undergo rapid cooling using liquid nitrogen or another cryogenic fluid subsequently. This cryogenic environment helps refine the microstructure and reduce grain size, enhancing the material’s strength, ductility, and toughness beyond what is achieved with conventional cold rolling, due to the even lower processing temperatures. In addition, it helps prevent defect formation and enhances the uniformity of the material. Ti cryogenic rolling is particularly advantageous for producing high-performance Ti alloys used in demanding applications such as aerospace, defense, and medical implants. This process can be applied to other metals and alloys to improve their mechanical properties.

Cryogenic rolling of CP-Ti, achieved by immersing samples in liquid nitrogen at −196 °C before rolling, significantly increases twinning formation, specifically tensile twinning on $\left\{10\bar{1}2\right\}$ and $\left\{11\bar{2}1\right\}$ and compressive twinning on $\left\{11\bar{2}2\right\}$ and $\left\{11\bar{2}4\right\}$. This increased twinning effectively inhibits slip along dislocations, enhancing the strength of the material. Many studies have confirmed that this process under cryogenic conditions promotes a refined microstructure, which plays a key role in the observed strength improvement.

**Figure 23:**
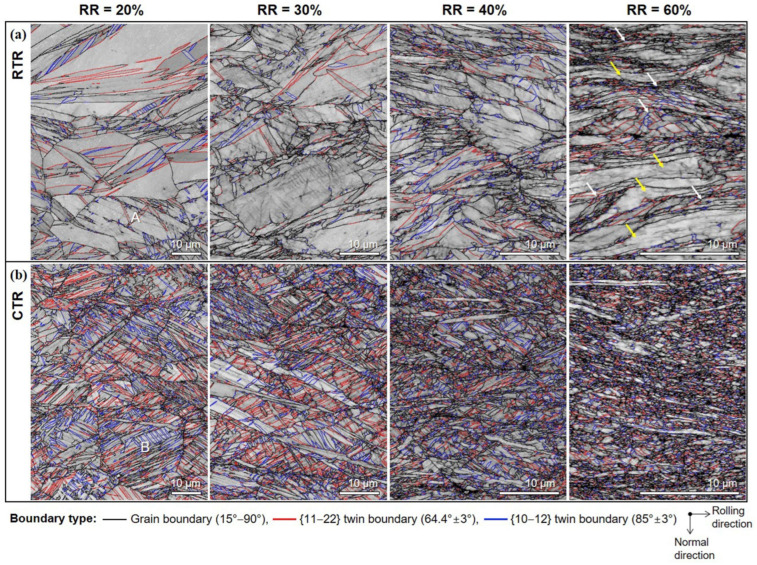
Grain boundary map of rolled Cp-Ti at a 20% to 60% reduction rate: (**a**) RTR and (**b**) CTR [88].

The research conducted by Won et al. applied cryogenic temperature rolling (CTR) to CP-Ti to study the differences in strength and microstructure compared to cold rolling [88]. They processed CP-Ti (grade 2) into a thickness of 4 mm, performing hot rolling at 700 °C with a reduction of less than 20% per pass and a final reduction rate of 85%. This was followed by a mill annealing process at 700 °C for 1 h. Afterward, the samples were subjected to cryogenic rolling, where they were immersed in liquid nitrogen at −196 °C for over 10 min at a reduction rate of less than 10% for each pass, leading to final reductions of 20%, 30%, 40%, 60%, and 75%. Figure 23 illustrates the microstructures of cold and cryogenic rolling at various reduction rates using EBSD analysis. As the reduction rate increases in both rolling processes, the occurrence of twins also increases. A comparison of the twin ratios in Figure 23a,b reveals that samples undergoing CTR have a significant number of twins at a reduction rate of 20%, whereas samples undergoing RTR have a significantly lower ratio at the same reduction rate.

**Figure 24:**
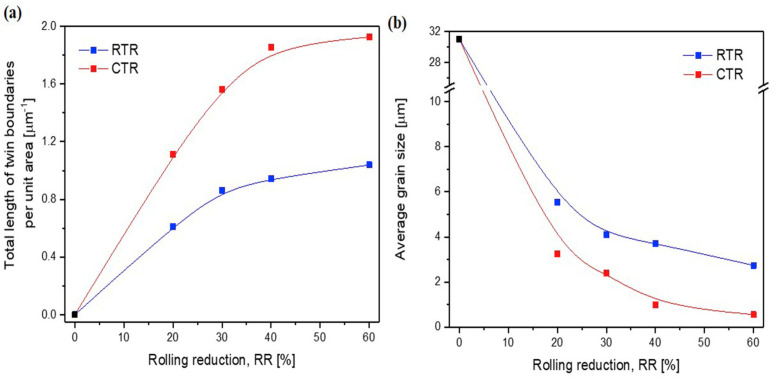
(**a**) Total length of twin boundaries per unit area and (**b**) average grain size represented according to the rolling reduction rate [88].

**Figure 25:**
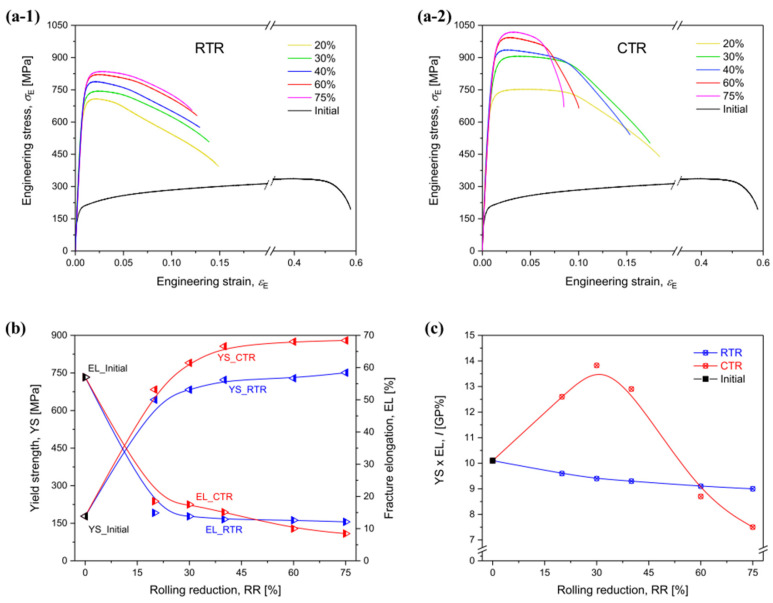
Tensile test results of initial vs. rolled materials: (**a-1**) engineering stress–strain curve of RTR, (**a-2**) engineering stress–strain curve of CTR (**b**), YS and elongation according to reduction rate, and (**c**) YS × elongation according to reduction rate [88].

Figure 24 shows the changes in the length of the twin boundaries and the average grain size in the two rolling processes according to the reduction rate. In the case of cryogenic rolling, as the reduction rate increases, the total length of the increased twin boundaries grows larger than that observed in cold rolling. Additionally, the average particle size is noted to be smaller, ranging from 1 to 4 µm, compared to 3 to 6 µm in cold rolling. Owing to these microstructural characteristics, Figure 25 illustrates the changes in YS and elongation according to the reduction rate for the two rolling processes. In Figure 25b, when CTR is at a reduction rate of 60%, the YS increases to 875 MPa. Simultaneously, as seen in Figure 25c, the elongation decreases. These experimental results demonstrate that, in the case of cryogenic rolling, an increase in the twin boundary length and ratio leads to a smaller average particle size, resulting in a significantly higher strength value compared to cold rolling.

**Figure 26:**
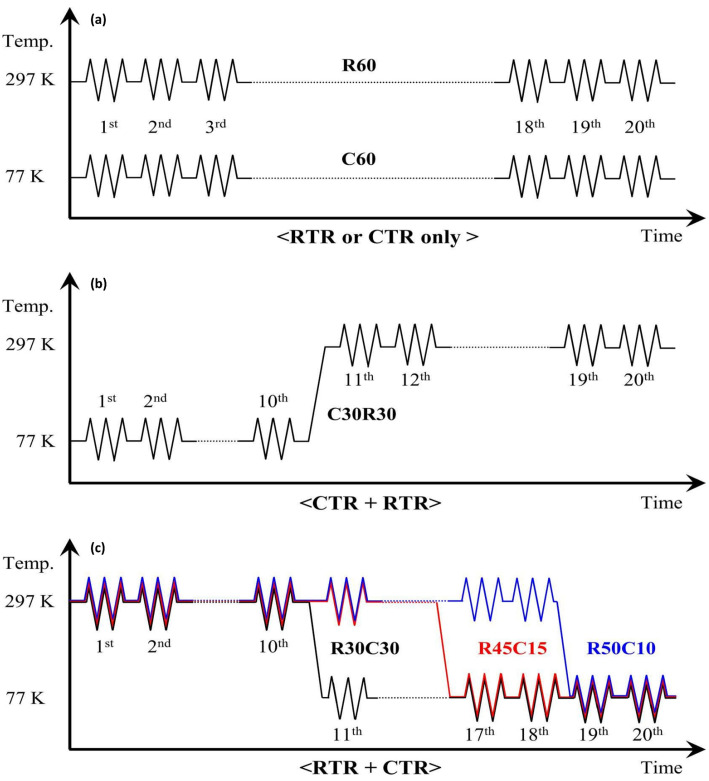
Schematics of rolling process for (**a**) CTR and RTR only; (**b**) RTR after CTR; (**c**) CTR after RTR [89].

Hong et al. developed a novel cryogenic rolling method that enhanced the strength of CP-Ti while reducing the number of cryogenic processing steps [89]. In this study, the rolled samples were cut into rectangular shapes with widths of 50 mm, lengths of 100 mm, and thicknesses of 5 mm. Twenty rolling processes were performed to produce sheets with a thickness of 2.0 mm, achieving a total area reduction of 60%. Each rolling process was performed using RTR (297 K), CTR (77 K), or a combination of both (Figure 26). To maintain the cryogenic state during CTR rolling, the samples were immersed in liquid nitrogen for more than 10 min. The team found that the order of RTR and CTR significantly affects the strength and that applying CTR to only a portion of the total area reduction can induce similar strengthening effects when applied to the total area reduction. The developed rolling method enabled significant strength enhancement while minimizing ductility reduction. This led to an excellent strength–ductility combination and improved cost efficiency by reducing the proportion of cryogenic treatment.

**Figure 27:**
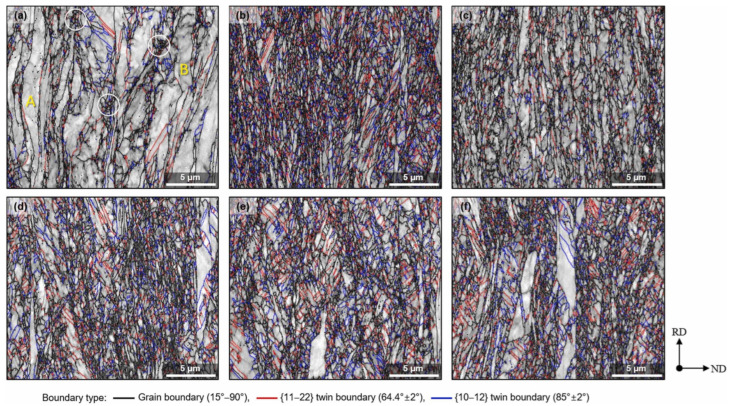
GB map of the rolled materials depending on different rolling processes: (**a**) R60, (**b**) C60, (**c**) C30R30, (**d**) R30C30, (**e**) R45C15, and (**f**) R50C10 [89].

The deformed microstructure is shown in Figure 27; the characteristics vary according to the rolling process. In R60, most grains were elongated along the RD, which is commonly observed in rolled materials. In this context, twinning was observed only in specific grains, where these grains showed fine isotropic division and grain refinement induced by twinning. Notably, the amount of twinning varied significantly across the grains, with some grains exhibiting minimal or no twinning and appearing to be elongated. Most grains with minimal or no twinning were oriented at Φ = 35–50°, indicating a direction unfavorable for twinning formation during rolling. These observations suggest that the twinning activity during RTR in pure Ti is highly sensitive to the grain orientation. Consequently, the elongated grains were surrounded by isotropic fine grains, forming a heterogeneous microstructure.

In contrast, the C60 material exhibited a distinctly different deformed microstructure. This material showed much more intense grain refinement induced by twinning than R60, with this effect occurring in most grains. Consequently, almost no elongated grains existed, and only isotropically refined grains were present, leading to a highly uniform microstructure. Unlike the R60 material, twinning was anticipated to form even in the C60 material grains with orientations unfavorable for twinning, suggesting that cryogenic deformation may have weakened the strong dependence of the grain orientation on twinning formation in CP-Ti.

In the C30R30 and R30C30 materials, significant grain refinement induced by twinning was also observed, resulting in more refined and uniform microstructures than those in the R60 material. However, the grain refinement induced by twinning in these two materials appeared to be less pronounced than that in the C60 material, likely because of the lower accumulated reduction (AR) applied during CTR in the C30R30 and R30C30 samples than in the C60 sample. The calculated average grain sizes were 0.84 µm for the C30R30 material and 0.75 µm for the R30C30 material, which were larger than those of the C60 material (0.55 µm) but smaller than those of the R60 material (2.02 µm). Similarly, strong grain refinement induced by twinning was observed in the R45C15 and R50C10 materials, with average grain sizes of 0.78 µm and 0.91 µm, respectively. In all the processed materials, the misorientation angles of the twinning boundaries were predominantly 64.4° and 85°, which correspond to the characteristic misorientation angles of tensile twinning systems $\left\{11\bar{2}1\right\}$ and $\left\{10\bar{1}2\right\}$, respectively. These results indicated that the $\left\{11\bar{2}1\right\}$ and $\left\{10\bar{1}2\right\}$ tensile twinning systems were primarily activated during rolling.

**Figure 28:**
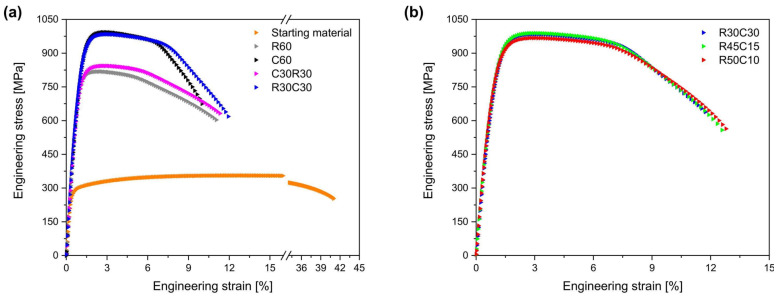
(**a**) Strain–stress curves of starting Ti, R60, C60, C30R30, and R30C30, and (**b**) R30C30, R45C15 and R50C10 Ti [89].

The strain–stress curves in Figure 28 indicate that the strength of the deformed material varies depending on the rolling process. The YS and UTS of the initial material are 280 and 356 MPa, respectively. The C60 material showed a substantial increase, with a YS of 825 MPa and a UTS of 993 MPa, representing a strength enhancement of 2.95 times and 2.79 times, respectively, compared to the initial material. Although R60 exhibited an increase in strength, it was lower than that of C60, whereas the strength of R30C30 was similar to that of C60. However, the C30R30 process resulted in less strength improvement than the C60 process, highlighting the impact of the order of RTR and CTR processes. The tensile properties of the R45C15 and R50C10 materials were also evaluated, and the R45C15 material exhibited YS and UTS values similar to those of the R30C30 material. Although it exhibited a slightly lower UTS, its YS was nearly identical. These findings suggest that even applying an AR of only 10% or 15% to CTR can achieve considerable strength enhancement.

**Figure 29:**
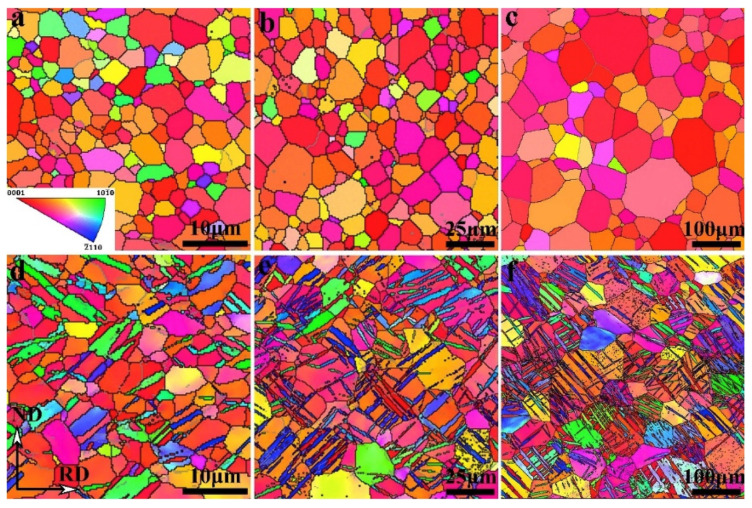
IPF map of annealed Ti samples after hot-rolling process: (**a**) grain size of 4 μm, (**b**) 10 μm, (**c**) 50 μm, and Ti samples subjected to cryogenic temperature rolling; (**d**) previous grain size of 4 μm, (**e**) 10 μm, and (**f**) 50 μm [90].

Li et al. investigated the influence of the differences in grain size after hot rolling on the twinning ratio when a Ti alloy with a composition of Ti-0.19Al-0.13V-0.147Fe-0.098Zr-0.033Cr was subjected to cryogenic rolling [90]. This alloy was initially processed by rolling at 700 °C with an 85% final reduction rate, followed by annealing at 700 °C for 1 h to prepare the initial sample. Subsequently, the sample was immersed in liquid nitrogen (−196 °C) for 10 min and then subjected to cryogenic rolling with a target reduction rate of 75%. Figure 29 visually presents the twinning ratios for different grain sizes (4 μm, 10 μm, and 50 μm) when cryogenic rolling was conducted at an 8% reduction rate. The highest twinning ratio was observed in Figure 29f, which exhibited the largest grain size.

**Figure 30:**
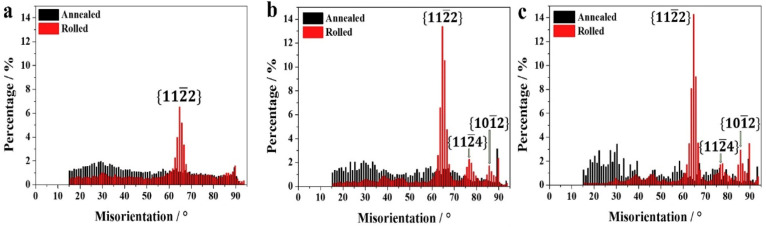
Comparison of percentage between annealed materials and rolled materials to misorientation, with an average grain size of (**a**) 4 μm, (**b**) 10 μm, and (**c**) 50 μm [90].

Figure 29 shows the distribution of the twinning ratios associated with the specific misorientation angles divided into pre- and post-rolling for extraction and extrusion. As observed, twinning is enhanced after rolling, with deformation twins forming to accommodate strain, which is reflected in the twinning ratios and misorientation angle distributions. As illustrated in Figure 30, significant twinning is observed after cryogenic rolling in the regions with the largest particle sizes. There are no misorientation peaks in annealed Ti, regardless of the average grain size. After the rolling process, a clear misorientation peak at around 65° is observed in Ti, with an average grain size of 4 μm, which corresponds to the $\left\{11\bar{2}2\right\}$ CT twinning system in HCP material. As the grain size increases, additional peaks at approximately 77° and 85° appear, corresponding to the $\left\{11\bar{2}4\right\}$ CT and $\left\{10\bar{1}2\right\}$ ET twinning systems, respectively. The intensity of the $\left\{11\bar{2}2\right\}$ CT peak is significantly higher than those of the $\left\{11\bar{2}4\right\}$ CT and $\left\{10\bar{1}2\right\}$ ET peaks. These findings indicate that the number of twinning systems increases with grain size, with the $\left\{11\bar{2}2\right\}$ CT system being the dominant one in Ti deformed at cryogenic temperatures. This increase in the twinning ratio led to an improvement in the YS from 450 MPa and the UTS from 381 MPa prior to rolling to 605 MPa and 525 MPa, respectively, after rolling, thereby demonstrating the effect of twinning. Consequently, while Won et al. focused on increasing the twinning ratio through cryogenic rolling to improve the strength [88], Huang et al. proved that particle size also acts as a factor influencing the formation of twinning [90].

Zhidong Chen et al. investigated the effects of strain rate on the formation of multimodal grain structures and the tensile behaviors of CP-Ti (Grade 2) [91]. In this study, CP-Ti sheets with an initial thickness of 2.5 mm were CTR to a final thickness of 0.75 mm, achieving a 70% thickness reduction and an equivalent strain of 1.39. The samples were immersed in liquid nitrogen before each rolling pass to ensure a cryogenic temperature of −196.15 °C. Rolling was conducted at strain rates ranging from 0.67 to 4.67 s^−1^, followed by annealing at 500 °C for 10 min in an argon atmosphere. Microstructural analysis revealed the formation of a multimodal grain structure consisting of nanoscale, ultrafine, and coarse grains. This structure resulted from the suppression of dynamic recovery and enhanced nucleation of recrystallization. The multimodal grain structure provided an excellent combination of strength and ductility, as tensile tests demonstrated improved strain-hardening behavior due to interactions among grains of varying sizes. This study highlighted the critical role of strain rate in controlling microstructural evolution during CTR and achieving desirable mechanical properties in CP-Ti.

**Figure 31:**
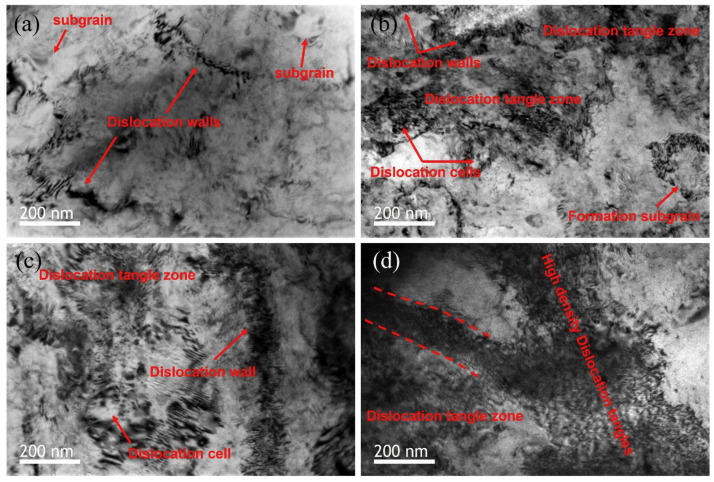
TEM images of CP-Ti after CTR at different strain rates: (**a**) 0.67 s^−1^, (**b**) 2.00 s^−1^, (**c**) 3.33 s^−1^, and (**d**) 4.67 s^−1^, illustrating the evolution of the microstructure with increasing strain rate [91].

Figure 31 illustrates the TEM (transmission electron microscope) images of cryo-rolled Ti at varying strain rates: (a) 0.67 s^−1^, (b) 2.00 s^−1^, (c) 3.33 s^−1^, and (d) 4.67 s^−1^. The microstructures of the four samples reveal fuzzy and poorly defined grain boundaries, a result of the intense plastic deformation. At a strain rate of 0.67 s^−1^, the original coarse grains fragment, with a limited number of dislocation walls forming alongside the emergence of small subgrains. The elongated dislocation walls are characterized by dense dislocation tangles, while the regions between these walls show a relatively lower dislocation density. This configuration enables the formation of tiny, dislocation-free subgrains (Figure 31a), indicating that dynamic recovery occurred during the rolling process. Sharp dislocation walls transform into subgrain boundaries through this recovery mechanism. As the strain rate increases to 2.00 s^−1^, the dislocation density rises significantly. Along with the dislocation walls, more regions of dislocation tangles appear, accompanied by the formation of dislocation cells. In the lower portion of Figure 31b, some dislocations tend to rearrange and cancel out, forming subgrain boundaries, indicating that subgrain formation remains active and recovery processes are ongoing at this strain rate. At a strain rate of 3.33 s^−1^ (Figure 31c), the microstructure primarily consists of dislocation walls and tangled dislocations, with only a few dislocation cells visible. No evidence of subgrains, either fully formed or in the process of formation, is observed, suggesting that dynamic recovery is nearly absent at this strain rate.

When the strain rate is increased to 4.67 s^−1^ (Figure 31d), high-density dislocation entanglements merge into the largest structures, with lath-like dislocations (approximately 100 nm in width) connecting these entangled regions. This indicates that dynamic recovery is effectively suppressed, resulting in a remarkably higher dislocation density within the sample. It is clear that higher strain rates lead to an increased dislocation density because rapid strain rates hinder dynamic recovery and promote dislocation multiplication. In the study, a typical microstructure characterized by disorganized, high-density dislocations interspersed with adjacent dislocation laths is observed under high-strain-rate CTR. Notably, the influence of strain rate on the microstructure is more pronounced during CTR compared to rolling at room temperature. This difference arises because stress levels during low-temperature deformation are considerably higher than those at room temperature, where deformation mechanisms are easier to activate. Consequently, low-temperature deformation promotes the generation and accumulation of dislocations, amplifying the effect of strain rate on the microstructure.

**Figure 32:**
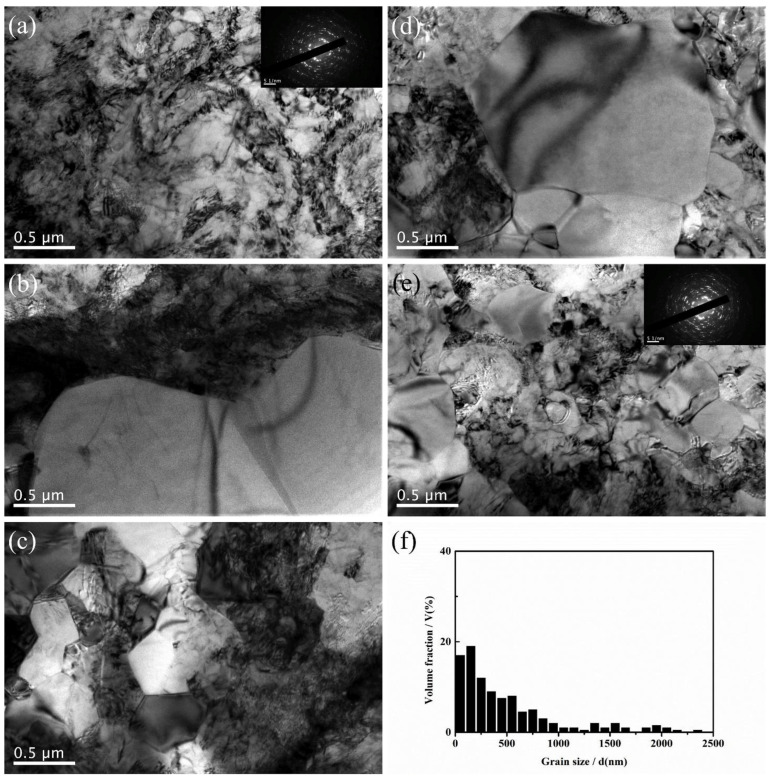
TEM images of cryo-rolled CP-Ti after annealing at 500 °C for 10 min at different strain rates: (**a**) 0.67 s^−1^, (**b**) 2.00 s^−1^, (**c**) 3.33 s^−1^, and (**d**,**e**) 4.67 s^−1^; (**e**) nanoscale and ultrafine grain region of (**d**); (**f**) statistical grain size distribution of cryo-rolled CP-Ti at 4.67 s^−1^ strain rate after annealing at 500 °C for 10 min [91].

Figure 32 presents TEM analysis of cryo-rolled Ti samples annealed at 500 °C for 10 min, revealing the evolution of the microstructure at various strain rates. At a strain rate of 0.67 s^−1^, the sample still exhibits a relatively high dislocation density, with no obvious recrystallized grains visible following annealing. The microstructure consists of a few nanoscale subgrains (Figure 32a), suggesting that partial recovery occurred during the annealing process. Selected area electron diffraction (SAED) patterns show diffraction spot circles with broken rings in the diffraction patterns (inset in Figure 32a), indicating the formation of subgrains bounded by LAGBs. At a strain rate of 2.00 s^−1^, some coarse grains with free dislocations begin to appear, while other areas still exhibit dislocation cells and subgrains with a high dislocation density (Figure 32b). At a strain rate of 3.33 s^−1^, the number of recrystallized grains increases (Figure 32c). The dislocation density varies significantly across the microstructure: Some grains are nearly free of dislocations, while others display dislocations aligned along subgrain boundaries or absorbed into these boundaries. Despite the observed recrystallization, the process remains incomplete, with un-recrystallized areas showing high dislocation densities. At a strain rate of 4.67 s^−1^, the microstructure is predominantly composed of nanoscale and ultrafine grains, with some coarse grains resulting from abnormal growth during recrystallization (Figure 32d,e). High-density dislocations still exist in certain regions. The diffraction pattern inset in Figure 32e demonstrates a high proportion of nanoscale and ultrafine grains with HAGBs. Statistical data in Figure 32f reveal a multimodal grain size distribution, with approximately 17% nanoscale grains, 70% ultrafine grains, and 13% coarse grains. These findings provide clear evidence of a diverse grain structure resulting from cryogenic rolling and annealing in the Ti sample.

**Figure 33:**
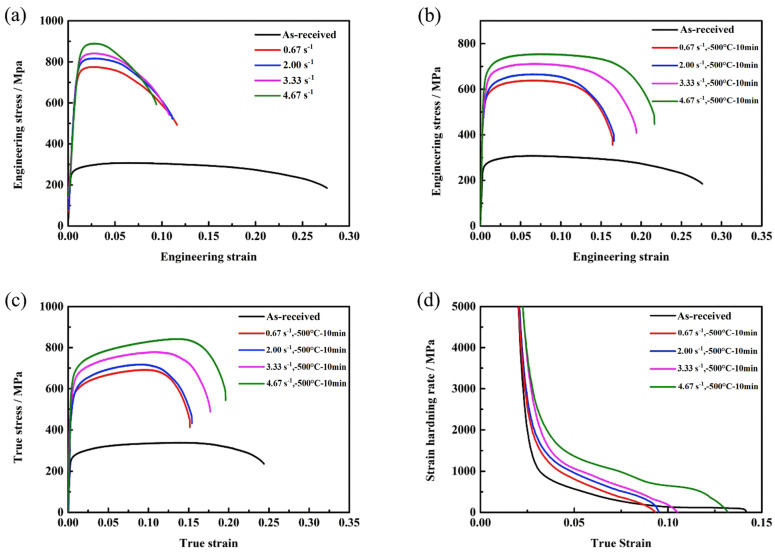
Tensile behavior of as-received Ti and cryo-rolled Ti at various strain rates before and after annealing at 500 °C for 10 min. (**a**) Stress–strain curves of as-received Ti and cryo-rolled Ti at different strain rates before annealing; (**b**) after annealing at 500 °C for 10 min. (**c**) True stress–strain curves, (**d**) strain-hardening rate curves for as-received Ti and cryo-rolled Ti at different strain rates after annealing at 500 °C for 10 min [91].

Figure 33 shows the tensile properties of as-received Ti and cryo-rolled Ti samples at different strain rates, both before and after annealing at 500 °C for 10 min. The UTS of all CTR samples significantly increases after the CTR process. As the strain rate increases from 0.67 s^−1^ to 4.67 s^−1^, the corresponding UTS rises from approximately 774 MPa to 889 MPa (Figure 33a). This increase in strength with higher strain rates is attributed to the enhanced dislocation density and grain refinement, suggesting that higher strain rates effectively improve the material’s strength. However, the elongation shows minimal variation across all samples, with typical brittle fracture behavior observed.

Following annealing at 500 °C for 10 min, the strength of all cryo-rolled-Ti samples decreases, while their ductility improves (Figure 33b). At the lowest strain rate of 0.67 s^−1^, the UTS decreases to around 638 MPa, while the uniform elongation increases to about 6.7% and the failure elongation rises to 16.4%. This enhanced ductility is attributed to the reduced dislocation density due to recovery during the annealing process compared to the as-received Ti. For the sample cryo-rolled at a strain rate of 2.00 s^−1^, the UTS and elongation are similar to those of the 0.67 s^−1^ sample, as recrystallization is limited and recovery dominates the microstructural evolution. Increasing the strain rate to 3.33 s^−1^ results in a UTS of around 711 MPa, with uniform elongation increasing to 6.8% and failure elongation rising significantly to 16.6%. These improvements in strength and ductility are due to the increased recrystallization, which forms a larger number of ultrafine grains and a higher fraction of HAGBs. At a strain rate of 4.67 s^−1^, UTS drops to approximately 753 MPa, while the uniform elongation increases to 8.0% and the failure elongation rises dramatically to 21.7%, indicating a combination of high strength and ductility. This combination is attributed to the multimodal grain structure, where nanoscale and ultrafine grains, along with dislocations, contribute to the high strength, and coarse grains promote good ductility.

To further investigate the ductility of the cryo-rolled samples after annealing, the true stress–strain curves are shown in Figure 33c, and the strain-hardening rate (defined as the rate of change of stress with respect to strain) versus true strain for the corresponding samples is presented in Figure 33d. The strain-hardening rates for all cryo-rolled samples after annealing are higher than those of the as-received Ti, owing to the dislocations introduced during the cryo-rolling process, which are consistent with conventional hardening mechanisms. The strain-hardening curves for the cryo-rolled samples at strain rates of 0.67 s^−1^, 2.00 s^−1^, and 3.33 s^−1^ remain nearly parallel during plastic deformation, with strain hardening increasing as the strain rate increases. The increase in strain-hardening capability contributes to the slight improvement in uniform elongation, which results from the increased recrystallization nucleation. For the sample cryo-rolled at 4.67 s^−1^, the annealed sample exhibits a notably high strain hardening, arising from the multimodal grain structure, leading to a more complex stress state and straining pattern during tensile deformation, as deformation is accommodated between grains of different sizes. During the tensile test, coarse grains embedded in a matrix of nanoscale and ultrafine grains deform first, followed by the finer grains, which are constrained by the coarse grains and later deform to accommodate the strain. This accommodation of strain results in dislocation blocking and piling up at the grain boundaries, generating long-range back stress. The back-stress hardening contributes to the high strain hardening observed in this sample. As local stresses accumulate at the grain boundaries, deformation crosses from coarse grains to the finer grains, forcing the finer grains to deform to accommodate the strain from neighboring coarse grains. This mechanism explains why the failure elongation for the cryo-rolled sample at 4.67 s^−1^ is significantly enhanced to 21.7%.

Based on these findings, the effects of cryogenic rolling on the mechanical properties and microstructures of Ti and its alloys were investigated. When CTR was applied to CP-Ti, an increase in the twinning ratio was observed, which corresponded to an enhancement in the strength. Furthermore, when cryogenic rolling was conducted based on the initial particle sizes, larger particles exhibited a higher twinning ratio. These results indicate that CTR is a promising method for controlling the microstructure and improving the mechanical properties of Ti alloys. Additionally, when comparing the effects of different strain rates, it was found that higher strain rates lead to more pronounced dislocation accumulation and grain refinement, further improving the mechanical properties. At higher strain rates, the microstructure consists of nanoscale and ultrafine grains, with high-density dislocations and higher twinning ratios. These findings suggest that adjusting the strain rate during cryogenic rolling can provide additional control over the microstructure and strength. Future research should explore various deformation conditions and examine the applicability of this method to other metals and alloys, thereby contributing to material development.

### 3.2. Classification of Rolling Processes by Direction

**Figure 34:**
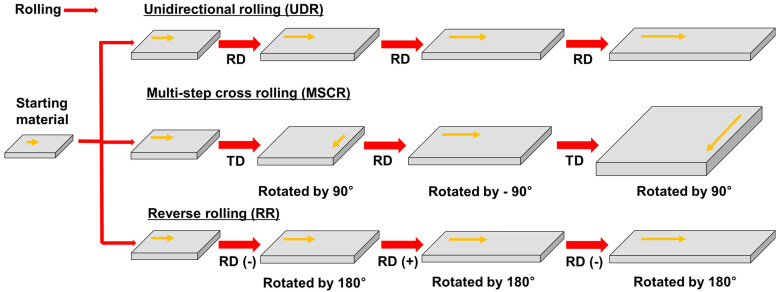
Schematic of UDR, MSCR, and RR rolling methods.

Cross-rolling is a specific rolling process that involves rotating the material at angles, typically 90°, during multiple passes, helping to create a more uniform microstructure. This approach mitigates the formation of preferred crystallographic orientations, thereby enhancing the isotropy of the material. In cross-rolling, the workpiece undergoes rolling at various angles, refining the grain structure and improving the mechanical properties, such as strength and ductility. This technique is particularly valuable in applications in which uniformity and resistance to deformation are crucial. This method aims to achieve a more balanced distribution of characteristics across a material, making it essential for tailoring materials for specific engineering applications. When a sample is fed to a rolling mill and subjected to multiple rolling passes, it can be categorized based on the rolling direction as UDR, MSCR, or RR. As illustrated in Figure 34, UDR entails continuing to roll the sample in the same direction as its initial entry into the mill. In contrast, MSCR involves rotating the sample by 90° during subsequent rolling passes after the initial input. In contrast, RR involves rotating the sample by 180° for each subsequent rolling process after the initial pass. Consequently, these directional processes lead to changes in the Ti microstructure.

**Figure 35:**
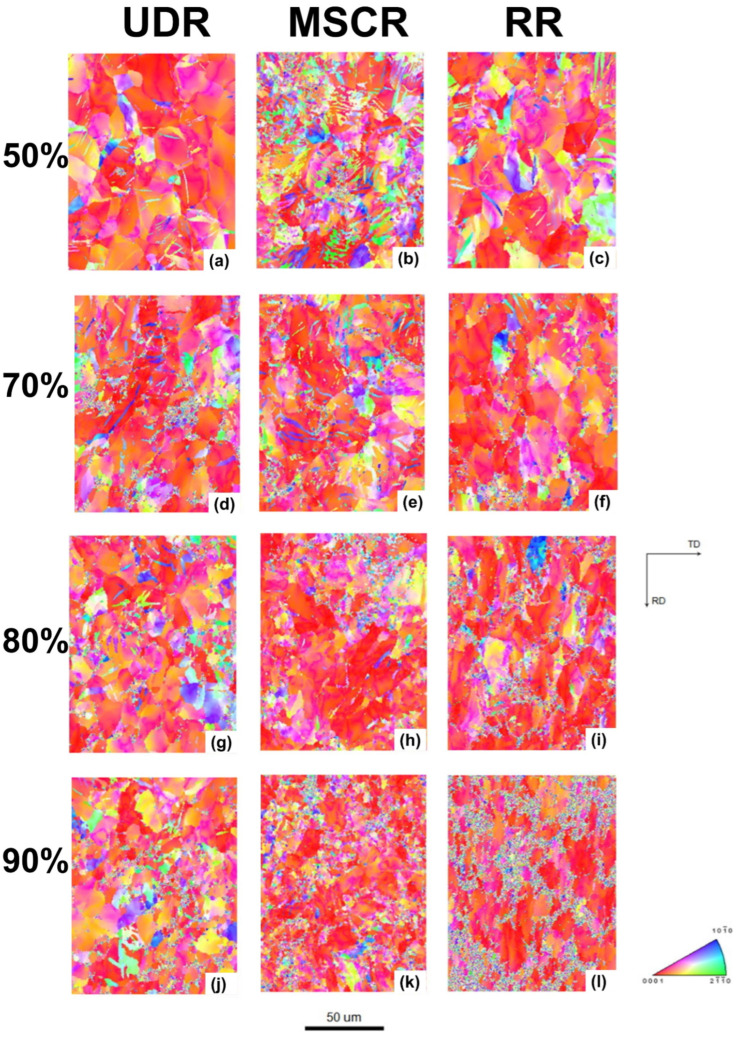
IPF maps of CP-Ti subjected to UDR, MSCR, and RR at different reduction percentages: (**a**–**c**) 50%, (**d**–**f**) 70%, (**g**–**i**) 80%, and (**j**–**l**) 90% [64].

Sahoo et al. investigated the microstructural characteristics of CP-Ti using hot-rolling processes, including UDR, MSCR, and RR, focusing on the effects of the rolling direction and reduction rate [64]. This study explains the deformation mechanisms that occur when various rolling methods are used. Figure 35 shows the IPF maps based on the reduction rate and rolling direction. Nearly isotropic grain structures were observed in all the samples, with no significant elongation, regardless of the deformation method or degree. The microstructural analysis confirmed the presence of twin boundaries within the samples. Notably, the samples processed via MSCR exhibited a significantly higher ratio of twin boundaries, particularly at reduction rates of 50% and 70%. By contrast, samples rolled using the UDR and RR methods showed notable twin boundary ratios only at a 50% reduction rate.

Two types of twin boundaries were observed in the samples. One was a tensile twin of the $\{1\bar{1}02\}<11\bar{2}0>$ type, whereas the other was a compressive twin of the $\left\{1\bar{2}12\right\}<1\bar{1}00>$ type. Analysis of the average grain size of the samples based on the reduction rate and various rolling methods revealed that in the cases of UDR and RR, the grain size tended to decrease as the rolling strain increased. In contrast, for the MSCR, the average grain size increased until a reduction rate of 80%, after which it decreased at a 90% reduction rate.

Particularly noteworthy is the potential for twinning in materials rolled at 600 °C via UDR, MSCR, and RR under a 50% reduction rate, with the process taking place over two stages. For the UDR, the shear plane where deformation occurs is indicated in red and purple in the schematic; as the reduction rate increases from 25% to 50%, the shear direction remains unchanged, maintaining a consistent possibility of twinning. This consistency arises because the twinning potential of the grains depends on their orientation when the material has a low stacking fault energy or a limited number of slip systems. In MSCR, the shear direction changes at each rolling stage, potentially increasing the incidence of twinning in the material. Conversely, for RR, the shear direction reverses in two consecutive rolling passes, which may enhance the twinning potential compared with UDR.

At higher reduction rates (from 80% to 90%), the formation of HAGBs varies depending on the hot-rolling method employed. In the UDR, the LAGBs formed after 80% reduction are expected to transition into HAGBs during the 90% reduction phase. In RR, a change in the rolling direction from 80% to 90% may lead to the complete annihilation of existing dislocations. In MSCR, while some dislocation annihilation occurs, newly formed shear planes can generate new dislocations that maintain a consistent ratio of LAGB throughout the rolling process. This analysis demonstrates the correlation between twinning, recrystallization, and grain-size variations in relation to the rolling direction and reduction rate in CP-Ti.

**Figure 36:**
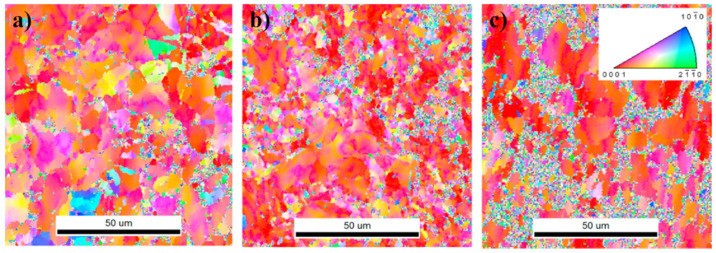
IPF maps of the hot-rolled CP-Ti samples depending on different strain pathways: (**a**) UDR, (**b**) MSCR, and (**c**) RR [65].

Additionally, Sahoo et al. built upon previous studies [64] to investigate the relationship between dislocation density and stored energy in CP-Ti as influenced by the UDR, MSCR, and RR methods [65]. Figure 36 shows IPF maps of the samples in the ND plane. The average grain size of the samples processed using the MSCR method was the smallest, followed by those of the RR- and UDR-processed samples. The average grain sizes measured were 4.74 μm for MSCR, 7.60 μm for RR, and 8.65 μm for UDR. Despite the variation in the deformation pathways, a strong basal-plane fiber texture was observed; however, the texture strength of the RR samples was higher than those of the MSCR and UDR samples.

**Figure 37:**
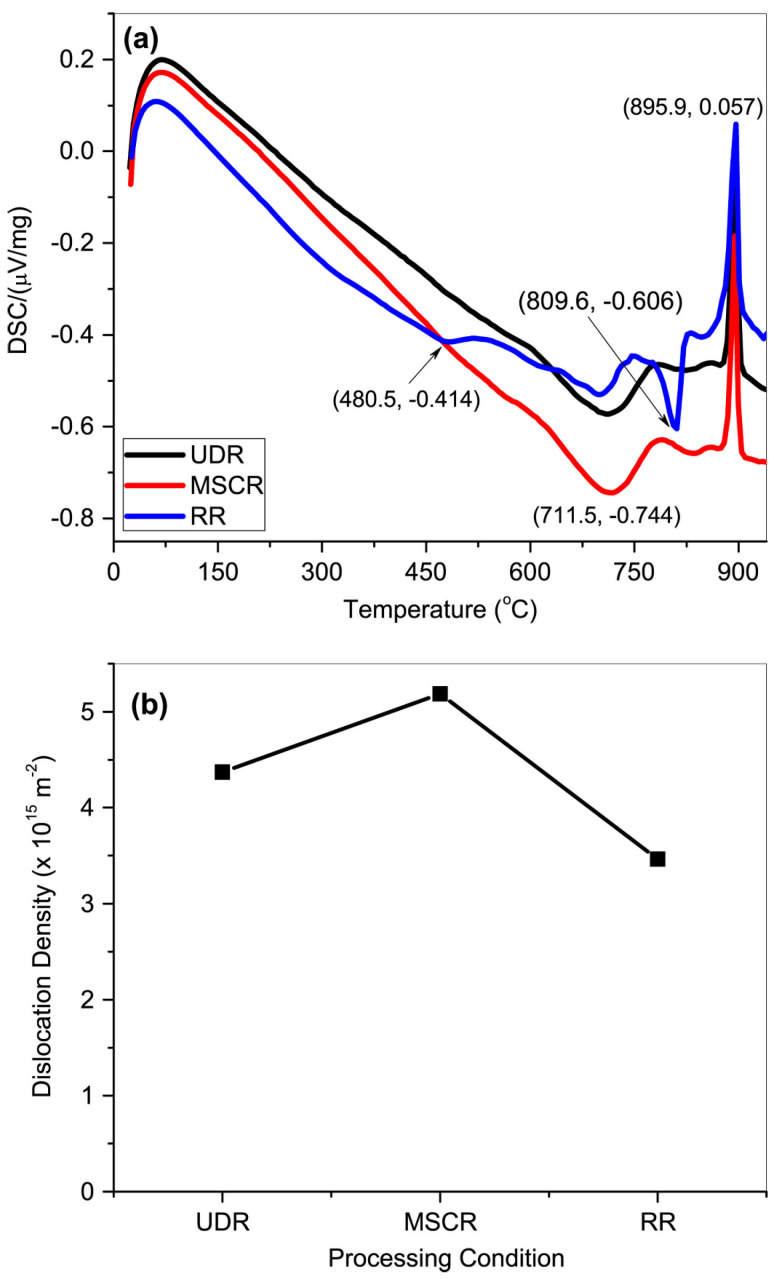
(**a**) DSC curves and (**b**) dislocation densities via XRD graphs for different processing conditions [65].

Figure 37 presents the differential scanning calorimetry (DSC) results and the dislocation density corresponding to each deformation pathway. A distinct exothermic peak related to recrystallization was observed only in the RR samples, whereas exothermic peaks corresponding to recovery were observed in all samples. The stored energy was the highest in the RR samples, followed by the MSCR and UDR samples. An endothermic peak observed at 894.6 °C in the samples was associated with the β-phase transformation. Figure 37b displays the estimated dislocation density based on the XRD line-broadening analysis according to the deformation pathways. The MSCR samples exhibited the highest dislocation density. As the dislocation density was measured based solely on the (0002) peak, a direct comparison between the DSC and XRD results was challenging.

**Figure 38:**
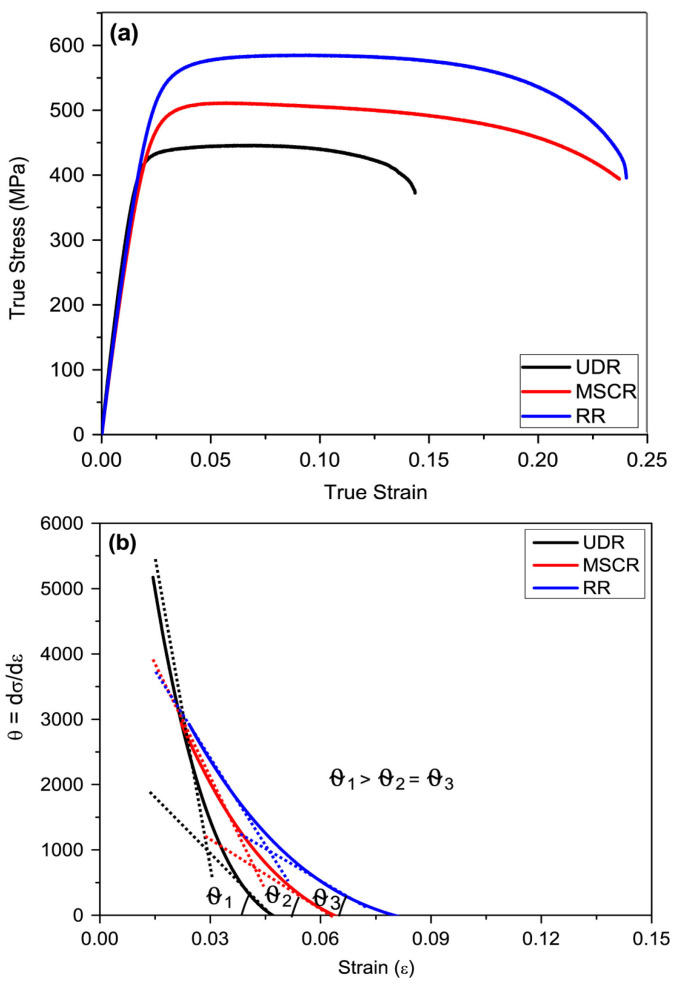
Stress–strain behavior of the samples as a function of strain paths: (**a**) true stress–strain plots and (**b**) hardening curves [65].

Figure 38 illustrates the stress–strain behavior of each hot-rolled sample. The RR samples exhibited higher YS, UTS, and ductility than the MSCR and UDR samples. Although the grain size of the RR samples was larger than that of the MSCR samples, the YS of the RR samples was higher, which may be attributed to the greater dislocation interactions. Ultimately, the RR samples demonstrated superior strength and ductility owing to their higher stored energy and increased dislocation density, attributable to variations in dislocation density and arrangement of grains.

The observed path dependence of the mechanical properties may be attributed to (1) differences in grain size or (2) differences in dislocation density and arrangement of grains. However, grain size was not a primary factor. Although the MSCR samples had smaller grain sizes than the RR samples, they exhibited lower YS and UTS. Therefore, it is expected that the dislocation density and arrangement of grains could explain the differences in the mechanical properties. This accumulation of dislocation is anticipated based on the stored energy (Figure 37a) and dislocation density (Figure 37b). The dislocation density in the basal plane was higher in the MSCR samples than that in the UDR and RR samples, whereas the RR samples potentially had more dislocations in the prism plane. The UDR samples exhibited lower dislocation densities in both the basal and prism planes than the MSCR and RR samples.

During the tensile tests, no hardening was observed with increasing strain, indicating that dislocation cross-slip from the prism plane to the basal plane was the only possibility for sustaining strain hardening. Dislocation cross-slip from the basal plane to the prism plane was unlikely because the basal plane was nearly parallel to the applied load direction. This difference in ductility may be attributed to the rate of dislocation cross-slip from the prism plane to the basal plane. This difference is also clearly shown in the graph of θ and ε (Figure 38b).

The higher YS of the RR samples compared with that of the MSCR samples may be due to more dislocation interactions occurring in the favorable prism plane of the RR samples. This study demonstrates that the RR samples exhibit better mechanical properties (YS and ductility) than the MSCR and UDR samples, primarily because of their higher stored energy, despite having larger grain sizes and similar textures. The rate of dislocation cross slip from the prism plane to the basal plane in both the RR and MSCR samples may contribute to the high ductility of these samples, whereas the high stored energy in the RR samples may be the reason for their greater strength.

**Figure 39:**
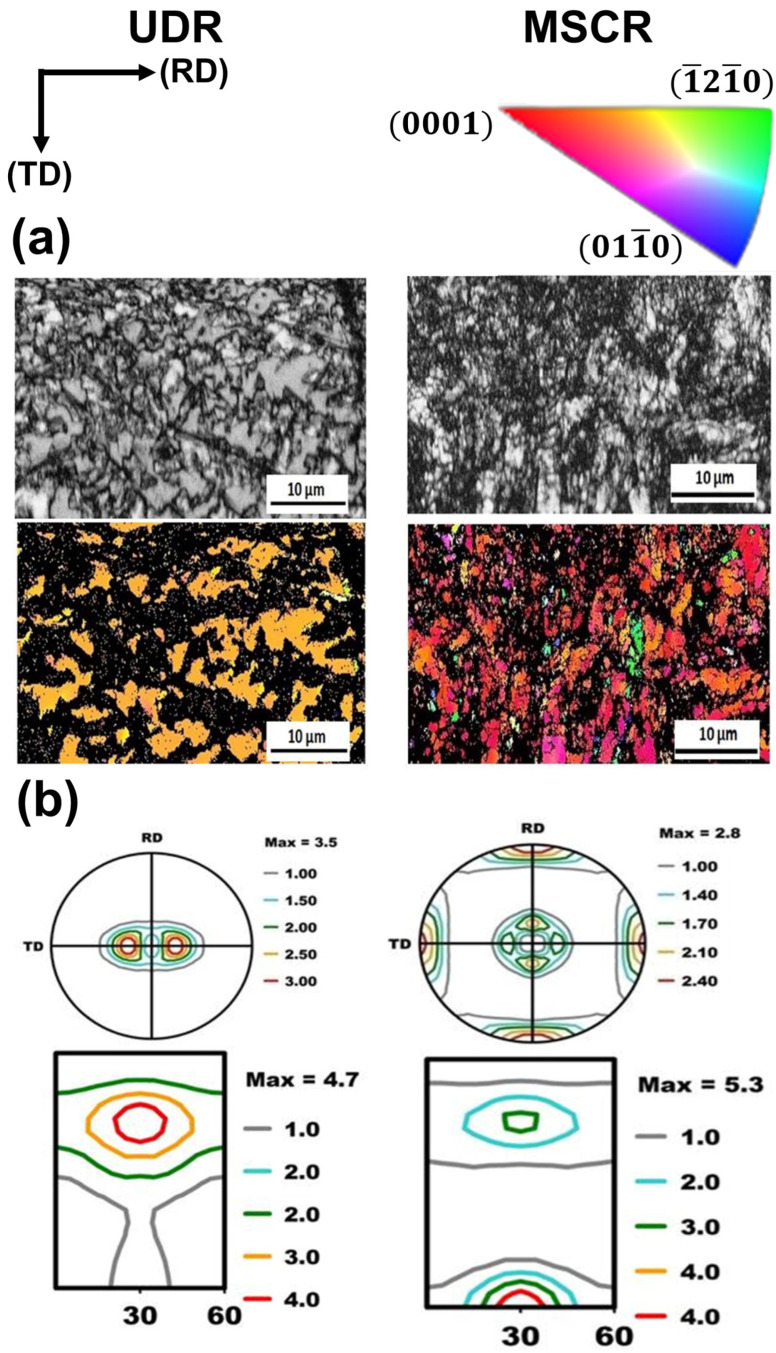
(**a**) EBSD map data and (**b**) pole figure (0002) and OD of F ϕ1 sections depending on different rolling directions (UDR and MSCR) [66].

Ghosh et al. studied the behavior of slip and twinning within a material by analyzing the texture evolution following UDR and MSCR under various heat-treatment temperature conditions [66]. This study focused on examining the texture evolution of CP-Ti during the cold rolling and annealing processes and analyzes the impact of texture on the mechanical properties. Figure 39a presents the pattern quality and crystal orientation maps of the cold-rolled UDR and MSCR samples. The very low pattern quality of the rolled samples indicates significant lattice distortion, whereas the IPF map displayed a high color gradient within the grains, representing the direction of misorientation due to dislocations. The $\left(0002\right)$ pole figure of the UDR sample revealed a TD-split basal texture, which is characteristic of cold-rolled HCP metals, with a c/a ratio of less than 1.633. The MSCR sample exhibited a nearly basal texture, with the basal plane aligned parallel to the normal plane of the sheet and spread in the RD and TD directions. The textural strength of the UDR sample was higher than that of the MSCR sample. Orientation distribution function map analysis shows that the maximum strength in the UDR sample occurred at ϕ1 = 0°, Φ = 37°, and ϕ2 = 30°, corresponding to the $\left(11\bar{2}4\right)<1\bar{1}00>$ direction. In contrast, the MSCR sample exhibited maximum strength at ϕ1 = 0°, Φ = 90°, and ϕ2 = 30°, which aligns with the $\left(11\bar{2}7\right)<1\bar{1}00>$ direction.

**Figure 40:**
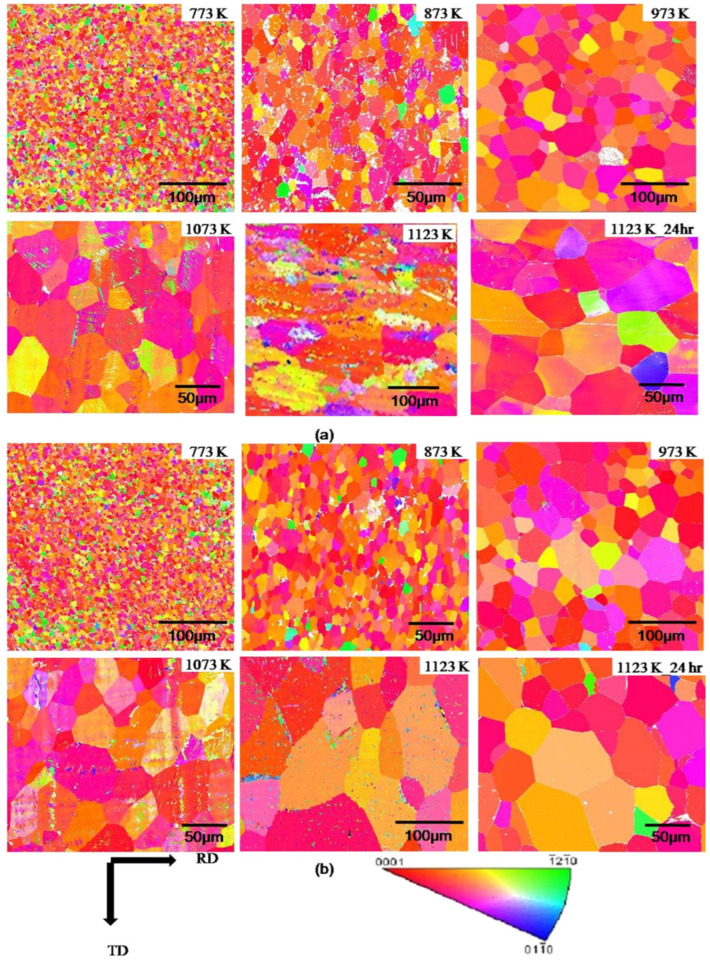
IPF maps showing recrystallized alpha grain structure of samples after annealing at 773–1123 K for 1 h and at 1123 K for 24 h in (**a**) UDR and (**b**) MSCR [66].

Figure 40 illustrates the changes in the microstructures of the UDR and MSCR under various heat-treatment conditions. At a recrystallization temperature of 973 K, the UDR sample showed an increase in texture strength, manifested by a decrease in the $<10\bar{2}0>$ texture component and an increase in $<10\bar{1}0>$ intensity. This indicates a transition in the recrystallization mechanism from nucleation to growth. As the temperature reached 1073 K, recrystallization occurred rapidly in the deformed regions, leading to grain growth as small grain fragments were absorbed. Notably, grains with a predominant $(0002)<10\bar{1}0>$ texture component exhibited texture separation in the TD direction, which played a crucial role in the mechanical slip of the UDR sample. In contrast, the MSCR sample, which had relatively low stored energy, underwent slower recrystallization, resulting in random nucleation and the emergence of two primary basal components. This contributed to a relatively weaker texture strength compared to that of the UDR sample.

**Figure 41:**
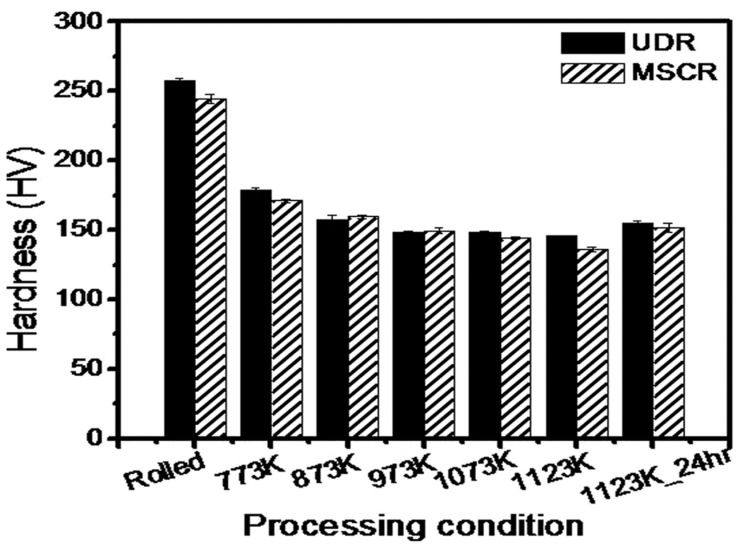
Hardness values of annealing samples depending on different temperature and rolling conditions (UDR, MSCR) [66].

As shown in Figure 41, the rolled samples exhibited significantly higher hardness than the annealed samples owing to their very high dislocation density. This increase in hardness is attributed to the greater local resistance of the deformed samples. In the annealed samples, hardness decreased as grain growth occurred. Although the grain sizes of the UDR and MSCR samples were similar at the same annealing temperature, the difference in hardness was thought to stem from the differences in texture. As mentioned earlier, the annealing texture varies based on the history of the deformation texture and is influenced by slip and twin systems that become active when adjacent grains have relatively different orientations. The difference in hardness between the two samples was not substantial; however, the variation in texture may have led to differences in the tensile properties of HCP materials, such as Ti.

In this study, the texture evolution and changes during recrystallization in Ti samples rolled using UDR and MSCR were observed. The UDR sample showed an increase in texture strength at high temperatures, forming a TD-reoriented texture that provided favorable conditions for mechanical slip. In contrast, the MSCR sample exhibited random nucleation and lower texture strength, displaying different characteristics during the recrystallization process compared to the UDR sample. Such research can provide important foundational data for improving the mechanical properties of Ti alloys through texture control.

## 4. Applications and Industrial Implications

The primary reason Ti-based products are used across various fields is due to their excellent corrosion resistance, combined with a low density of approximately 4.5 g/cm^3^ (0.16 lb/in^3^) and high strength [92]. Ti is categorized into several grades, each of which exhibits unique physical and mechanical properties based on its specific alloy composition. Grades 1–4 are classified as CP-Ti, and their compositions are listed in Table 2. CP-Ti (with a purity of over 99%) is a metal that exhibits low to moderate strength, making it unsuitable for high-load environments such as aircraft structures or engines. The YS of high-purity Ti ranges from 170 to 480 MPa, which is insufficient for aerospace structures.

According to the ASTM standards, CP-Ti is classified by a numbering system in the U.S., with designations such as Grade 1 and Grade 2; this classification is adopted in only a few countries. Although the tensile strength of CP-Ti can exceed 450 MPa, its specific strength is lower than that of Al alloys owing to the density differences. After refinement from rutile ore, CP-Ti retains a small amount of impurities, which mainly consist of iron and atomic oxygen. These impurities enhance strength and hardness through solid-solution hardening. For instance, ultra-pure Ti with an oxygen content of less than 0.01% exhibits a UTS of approximately 250 MPa, whereas an increase in oxygen content to 0.2–0.4% results in strengths of 300–450 MPa. However, this method of strength enhancement using impurities is undesirable because it significantly reduces the ductility, thermal stability, and creep resistance.

Although CP-Ti is rarely used in aircraft, its ability to maintain its strength and ductility at cryogenic temperatures makes it suitable for low-temperature applications. CP-Ti plays a crucial role, particularly in applications like liquid hydrogen storage tanks in spacecraft, as liquid hydrogen must be stored at temperatures below −210 °C, where Ti provides excellent strength and toughness.

From Grade 5 onwards, alloying elements have been added to Ti to enhance its mechanical strength (Table 3). Grade 5, or Ti-6Al-4V, is an alloy that includes Al and vanadium, featuring a mixture of α and β phases, known for its excellent strength and heat resistance in various applications, including aircraft and automotive components. Grade 7 contains a small amount of palladium added to Grade 2, providing exceptional corrosion resistance and making it suitable for chemical equipment. Grade 9, designated as Ti-3Al-2.5V, has a lower strength than Grade 5 but is utilized in heat exchangers and aircraft parts because of its excellent formability and thermal properties. Grade 11, which includes a small amount of palladium added to Grade 1, is suitable for deep drawing processes and exhibits excellent corrosion resistance in acidic environments.

The grades of Ti are selected according to their specific characteristics and applications, making them valuable in industries requiring high strength and durability. The minimum YS of Ti varies by grade, and the selected grade is influenced by the content of impurities, such as iron, nitrogen, carbon, oxygen, hydrogen, and silicon. As the impurity content increases, the grade tends to rise, leading to a decrease in machinability while increasing strength and hardness. The YS of CP-Ti ranges from approximately 170 MPa (Grade 1) to 485 MPa (Grade 4), and the tensile strength varies from approximately 240 MPa (Grade 1) to 550 MPa (Grade 4). In contrast, the structural Ti alloy products exhibit a YS of approximately 1100 MPa (160 ksi). The work-hardening effect of Ti can be alleviated through annealing, and the mechanical properties of pure Ti can be adjusted through rolling [35,93,94,95].

### 4.1. High-Strength and Lightweight Structural Material

The design and development of lightweight, high-strength structural materials remain a continuous goal in the field of materials research. Ti is a well-known high-performance structural material suitable for various applications owing to its light weight. Ti possesses high specific strength and excellent corrosion resistance, and these characteristics are maintained even at elevated temperatures.

**Figure 42:**
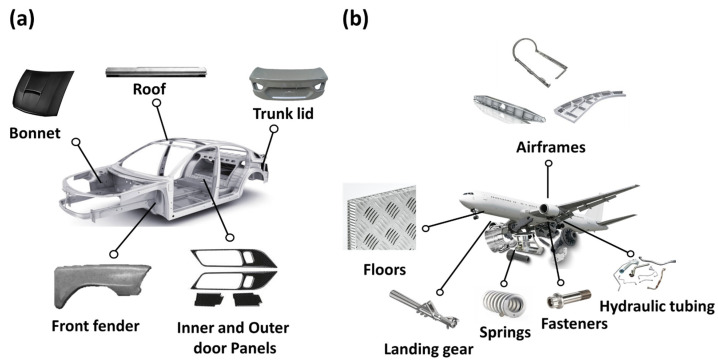
Main applications of Ti and its alloys after rolling process: (**a**) automotive and (**b**) aerospace.

Several methods can be employed to enhance the mechanical properties of Ti, including compositional design, processing techniques, and heat treatment. Through composition design, Ti alloys of α and β phases have been developed. By adjusting its microstructure using different processing methods and subsequent heat treatments, Ti can achieve high strength and ductility, making it applicable in various industries (Figure 42).

As the demand for lightweight electric and hydrogen vehicles continues to increase, reducing the weight of body panels (such as roofs, hoods, and doors) is becoming increasingly important because of their considerable mass proportion in automobiles. Specifically, lightweighting the roof, which is positioned at the top of the vehicle, can lower the center of gravity and enhance the dynamic performance.

In automobile manufacturing, the roof is typically integrated into a steel body frame and subjected to a painting process. During the painting process, the paint is heated to approximately 170 °C, which can lead to deformation if the coefficient of thermal expansion is high. The coefficient of thermal expansion for Ti is 8.4 × 10^−6^ (K), which is relatively low compared to steel’s 12 × 10^−6^ (K). In addition, Ti has an excellent strength-to-weight ratio that allows for weight reduction while maintaining rigidity. This facilitates lightweighting without compromising the durability and safety of the vehicles.

Therefore, applying Ti to vehicle roofs through rolling processes can enhance the strength while suppressing thermal distortion, owing to its low density and low coefficient of thermal expansion. Owing to its unique light weight and durable properties, Ti can play a crucial role in improving vehicle performance. Table 4 illustrates the applications of Ti in automotive components. CP-Ti is primarily used for its corrosion resistance, whereas Ti alloys are employed to enhance its mechanical properties [96,97].

The aerospace sector is one of the primary fields in which Ti materials are applied, accounting for 36% of engine systems and 7% of aircraft fuselage systems. In the United States, approximately 70–80% of the Ti demand arises from the aerospace industry, with the remainder used for industrial purposes. The main reasons for using Ti in aerospace applications are its low weight, space efficiency, heat resistance at operational temperatures, and excellent corrosion resistance. Owing to these properties, Ti has become an important material for replacing traditional steel or Al alloys.

In particular, Ti alloys offer higher strength than Al alloys, allowing the design of smaller components that can withstand the same loads, which is advantageous for weight reduction. Additionally, Ti alloys are more suitable for environments that exceed the temperature thresholds of Al alloys. For example, the high-temperature resistance of Ti is beneficial for applications such as nacelles, auxiliary power units, and wing de-icing systems. The use of Ti in the landing gear beams of the Boeing 747 and 757 exemplifies how Ti can address space constraints. Although Al 7075 is a cost-effective alloy, the size of the components required to support the necessary loads can be too large for confined spaces, leading to the use of Ti alloys.

In most cases, Ti provides excellent corrosion resistance, making painting unnecessary. However, when in contact with Al or low-alloy steel, painting may be necessary to prevent galvanic corrosion owing to the potential difference when exposed to the same electrolyte. In this regard, Ti demonstrates superior durability even in highly corrosive environments, such as structural supports in the lower areas of kitchens and bathrooms in aircraft. Table 5 lists various aircraft components with different alloy compositions [10,98,99].

Ti’s suitability as a lightweight, high-strength structural material is summarized in Table 6. Ti provides high specific strength and excellent corrosion resistance, even at elevated temperatures, making it ideal for automotive and aerospace applications. For automotive applications, Ti’s low thermal expansion coefficient (8.4 × 10^−6^ K) enables weight reduction in vehicle components, such as roofs, without compromising durability and rigidity, thereby enhancing dynamic performance. In aerospace, Ti alloys are widely utilized in components like nacelles, auxiliary power units, and landing gear beams due to their high strength-to-weight ratio and superior heat resistance compared to Al alloys. However, there are challenges in adopting Ti as a structural material. The high cost of Ti materials and complex manufacturing processes (e.g., rolling, forming) limit their use in cost-sensitive industries. Additionally, optimizing processing methods to achieve a balance between strength, ductility, and corrosion resistance while maintaining costs efficiency remains a significant challenge. Furthermore, achieving the right balance among weight reduction, strength, formability, and manufacturing feasibility continues to require ongoing development.

### 4.2. Ti Sputtering Targets for Quality Thin Films

**Figure 43:**
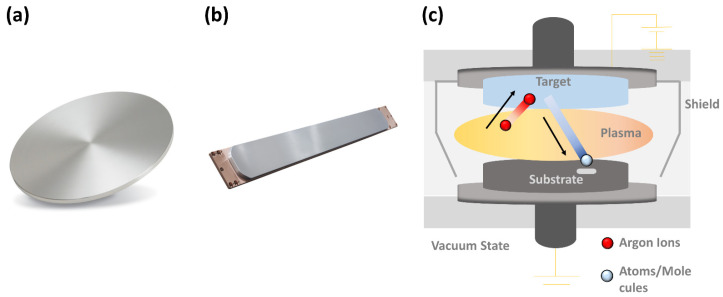
Ti sputter targets for (**a**) a semiconductor and (**b**) a display after the rolling process. (**c**) Schematic diagram of the sputtering process.

Sputtering targets are materials used in physical vapor deposition (PVD) to form thin films. In this process, the sputtering target begins in the solid state and is separated into small particles by gas ions, which are then sprayed onto the substrate surface. This method results in the formation of uniform thin films that are widely utilized in various industries, including semiconductors, displays, and solar panels (Figure 43) [100]. Among these, Ti sputtering targets play a crucial role because they belong to the Group IV transition metals, offering high mechanical properties, low density, excellent corrosion resistance, and high adhesion. In the semiconductor industry, Ti is primarily used in dynamic random-access memory (D-RAM) and logic integrated circuits, whereas in the display sector, it is employed in the manufacturing of OLED 6G and LCD 8G panels. Each industry has specific requirements for Ti targets, particularly in the field of integrated circuits, where characteristics such as high purity, fine-grain structure, and precise dimensional accuracy are essential [101]. The manufacturing process for sputtering targets includes casting (or powder processing), extrusion, hot pressing or rolling, cold pressing or rolling, heat treatment (recrystallization), and flattening. Among these processes, rolling is crucial for controlling the microstructure and achieving a uniform grain size.

Some properties of sputtering targets significantly affect the performance and characteristics of thin films during the deposition process. When thin films are deposited onto substrates using direct-current (DC) magnetron sputtering, the grain size of the target plays a critical role in the sputtering yield, particle size, and magnetic properties of the thin film. Targets with smaller grain sizes tend to exhibit higher sputtering yields and form thin films with larger particles [102]. Moreover, the purity and microstructure of the sputtering targets have a profound impact on the performance of the produced thin films [103]. High-purity sputtering targets, when annealed under an argon atmosphere at 700 °C, demonstrate excellent crystallinity and uniformly equiaxed grains. This results in deposited thin films exhibiting smoother surfaces, uniformly distributed fine particles, increased film thickness, lower surface roughness, and lower resistivity. In addition, in sputtering processes utilizing high-purity targets, a decreasing deposition rate over time is observed that is correlated with an increase in film thickness. Therefore, the grain size and microstructure of the sputtering targets have a significant impact on the sputtering yield, the microstructure of the films, and consequently, the physical, electrical, and magnetic properties of the films. By adjusting the various rolling process conditions, the microstructure of Ti-rolled products can be controlled, which in turn allows for the regulation of the grain size and structural properties of the targets. For example, high-purity Ti targets that are annealed at 700 °C exhibit a more uniform equiaxed crystal structure. This results in the deposition of Ti films with smoother surfaces, a uniform particle distribution, lower surface roughness, and reduced resistivity. Controlling the microstructure through rolling processes significantly affects not only the sputtering yield but also the performance of the deposited films. Thus, the Ti rolling technology plays a crucial role in advanced industries requiring high performance.

Ti sputtering targets play a key role in producing high-quality thin films for applications in semiconductors, displays, and solar panels, as summarized in Table 7. These targets provide excellent mechanical properties, low density, and corrosion resistance, which are essential for achieving high-quality thin films. Controlling the rolling process is crucial for optimizing the microstructure of Ti sputtering targets, and smaller grain sizes result in higher sputtering yields and improved film quality. Furthermore, high-purity Ti sputtering targets, when annealed at 700 °C, improve the crystallinity of the films, creating equiaxed grain structures that enhance the electrical and magnetic properties of the films. Despite these achievements, challenges remain in optimizing Ti sputtering targets for thin film production. Achieving the desired grain size and uniformity requires precise control over rolling and post-processing treatments. Balancing sputtering yield with film quality, such as surface roughness and film thickness, demands careful optimization of processing parameters. Additionally, maintaining high purity in the sputtering targets while managing costs remains a significant challenge in industries requiring high-quality thin films.

## 5. Conclusions

This study comprehensively reviewed the rolling technologies applied to Ti and its alloys, as well as texture analysis techniques. Various rolling processes aimed at analyzing the microstructure of Ti and enhancing its mechanical properties have been examined, confirming that these processes can significantly improve the mechanical properties by adjusting the grain size and texture of Ti. Both hot- and cold-rolling processes for Ti alloys play crucial roles in improving the mechanical strength, ductility, and durability by controlling the microstructure in distinct ways. Hot rolling can enhance the mechanical properties by reorganizing the crystal structure of the material at high temperatures, whereas cold rolling is effective in increasing the strength through the effective strain-hardening effect at room temperature. These rolling processes provide essential foundational data for elucidating the correlation between the microstructure and mechanical properties of Ti, and optimizing the processing conditions is vital for determining the quality and performance of the final products.

Texture analysis techniques play a significant role in understanding the complex relationship between the crystallographic structure and the mechanical properties of Ti. By utilizing XRD, EBSD, and mechanical property analysis methods, the crystallographic texture of Ti can be finely analyzed, allowing the identification of variations in mechanical properties under various processing conditions. The information obtained from texture analysis is essential for quantitatively evaluating the effects of the rolling processes and providing critical data for process adjustments aimed at improving specific mechanical properties. Additionally, texture analysis offers valuable insights into the mechanisms of plastic deformation during rolling processes. For instance, understanding the crystallographic texture variations induced by strain deformation is essential for predicting and improving mechanical properties. These analytical techniques serve as indispensable tools for enhancing the mechanical properties of Ti, enabling more precise control of its characteristics when combined with plastic deformation through rolling processes.

From an application perspective, the rolling and texture analysis techniques discussed in this study can significantly contribute to enhancing the performance of Ti and its alloys in various industrial sectors. For instance, in high-performance fields, such as aerospace, automotive, and medical devices, improving the strength and durability of Ti plays a crucial role in increasing the reliability and lifespan of components. Moreover, the rolling processes of Ti in advanced electronic devices and semiconductor industries help ensure that cutting-edge electronic materials exhibit stable performances. These technical characteristics have expanded the commercialization and industrial applicability of Ti, enabling innovative applications of Ti-based materials.

In conclusion, research on the control of the microstructure and enhancement of the mechanical properties of Ti and Ti alloys should extend beyond mere improvements in the rolling processes. It must encompass various research areas such as new alloy design, process optimization, and the introduction of advanced analytical techniques. This approach maximizes the usability of Ti and strengthens its competitiveness for industrial applications. Future research should focus not only on optimizing rolling conditions but also on elucidating the fundamental mechanisms of plastic deformation, such as twinning and slip, that occur during these processes. A deeper understanding of these mechanisms could enable innovative approaches to engineering the microstructure and enhancing specific properties of Ti and its alloys. Furthermore, future studies should explore experimental methods for controlling the microstructure of Ti and enhancing its mechanical properties. The rolling processes and texture analysis techniques presented in this paper provide essential foundational data for analyzing the microstructure and improving the mechanical properties of Ti and its alloys. This is expected to significantly contribute to innovative research and development in the future.

## Figures and Tables

**Table 1:** An overview of the advantages and disadvantages of various rolling methods and their effects on microstructure and texture.

Rolling Method	Microstructure and Texture Properties	Advantages and Disadvantages
Cold rolling	Stronger crystallographic texture, finer grains through work hardening	Improves strength, hardness, and surface finish Increases residual stress, limiting formability More precise thickness control Limited ductility due to strain hardening
Hot rolling	Formation of random texture with fine grains through recrystallization	Reduces residual stresses Limited precision and flatness Easier to process large reductions Can result in surface oxidation
Cryo-rolling	Promotes twinning, finer grains, and enhanced texture uniformity	Improves strength, ductility, and toughness Complex process with high cooling requirements Beneficial for high-performance applications Requires specialized equipment
UDR	Provides homogeneous grain structure along rolling direction, and textures align with the rolling direction	Suitable for products needing directional properties Lack of uniformity in other directions
MSCR	Promotes uniform deformation in multiple directions and results in more isotropic texture	Improves uniformity of microstructure Process complexity increases with each step
RR	Reduces residual stresses through repeated deformation and more uniform texture distribution across material surface	Improves surface quality and material uniformity May not be ideal for high thickness reduction

**Table 2:** Chemical composition and mechanical properties of CP-Ti according to ASTM standards.

Grade	Chemical Composition (Max, %)					Mechanical Properties		
	N	C	H	O	Fe	Tensile Strength (UTS/MPa)	$\mathrm{YS}\;(\sigma_{y}$/MPa)	Elongation (%)
Grade 1	0.03	0.10	0.015	0.18	0.20	240	170–310	25
Grade 2	0.03	0.10	0.015	0.25	0.30	340	275–450	20
Grade 3	0.05	0.10	0.015	0.35	0.30	450	380–550	18
Grade 4	0.05	0.10	0.015	0.40	0.50	550	485–655	15

**Table 3:** Chemical composition and mechanical properties of Ti alloy according to ASTM standards.

Grade	Chemical Composition (Max, %)								Mechanical Properties		
	N	C	H	Fe	O	Al	V	Pd	Tensile Strength (UTS/MPa)	YS $(\sigma_{y}$/MPa)	Elongation (%)
Grade 5	0.05	0.10	0.015	0.40	0.20	6.7	4.5	-	895	830	10
Grade 7	0.03	0.10	0.015	0.30	0.30	-	-	0.25	345	275–450	20
Grade 9	0.03	0.10	0.015	0.25	0.30	3	2.5	-	690	620	15
Grade 11	0.03	0.08	0.015	0.20	0.30	-		0.25	240	170–310	24

**Table 4:** Applications of Ti in various automotive components.

Ti Components	Automotive Parts
CP-Ti (Grade 1)	The outer shell of the muffler
CP-Ti (Grade 2)	Heat shields, exhaust systems
CP-Ti (Grade 3)	Door beams
CP-Ti (Grade 4)	Body panels, car bodies
Ti-6Al-4V	Body panels, car bodies, bumpers, axle suspension, crash clamps, suspension springs
Ti-5Al-2.5Sn	Exhaust systems
Ti-6.8Mo-4.5Fe-1.5Al	Suspension springs
Ti-10V-2Fe-3A	High-strength performance components

**Table 5:** Applications of Ti in various aerospace components.

Ti Components	Aerospace Parts
CP-Ti	Floors
Ti-3Al-2.5V	Hydraulic tubing
Ti-10V-2Fe-3Al	Landing gear
Ti-6Al4V	Window frames
Ti-15V-3Al-3Cr-3Sn	Springs, landing gear, plate and airframe castings
Ti-13V-11Cr-3Al	Airframe, landing gear, springs
Ti-6V-6Mo-5.7Fe-2.7Al	Fasteners

**Table 6:** Principal achievements and challenges in the high strength and lightweight structural materials of Ti rolling.

Section	Principal Achievements	Challenges
Automotive	High specific strength and corrosion resistance Low thermal expansion coefficient (8.4 × 10^−6^ K) Weigh reduction for vehicle components Maintains durability and rigidity despite weight reduction	High material cost Complex manufacturing Processing challenges Weight-to-strength trade-off
Aerospace	High specific strength and corrosion resistance High strength-to-weight ratio Superior heat resistance	

**Table 7:** Principal achievements and challenges in Ti sputtering targets for high-quality thin films.

Principal Achievements	Challenges
Ti sputtering targets enable high-quality films for semiconductors, displays, and solar panels. They offer excellent mechanical properties, low density, and corrosion resistance. Controlled rolling optimizes microstructure for better film quality. Smaller grain sizes increase sputtering yields and improve film properties. High-purity targets annealed at 700 °C enhance film crystallinity. They promote equiaxed grain structures for uniform properties. They improve the electrical and magnetic characteristics of thin films.	Precise rolling and post-processing optimize grain size and uniformity. Balancing sputtering yield and film quality like surface roughness and thickness is a challenge. Adjustments are critical to achieving optimal film properties. Ensuring high purity in sputtering targets for quality thin films is a challenge. Managing costs while maintaining purity remains difficult.

## Data Availability

No new data were created or analyzed in this study. Data sharing is not applicable to this article.
